# Rapid Enzymatic Detection of Shiga-Toxin-Producing *E. coli* Using Fluorescence-Labeled Oligonucleotide Substrates

**DOI:** 10.1021/acsinfecdis.4c00221

**Published:** 2024-11-22

**Authors:** Isabell Ramming, Christina Lang, Samuel Hauf, Maren Krüger, Sylvia Worbs, Carsten Peukert, Angelika Fruth, Brigitte G. Dorner, Mark Brönstrup, Antje Flieger

**Affiliations:** † Department for Infectious Diseases, Division of Enteropathogenic Bacteria and Legionella (FG11), National Reference Centre for Salmonella and other Enteric Bacterial Pathogens, Robert Koch Institute, 38855 Wernigerode, Germany; ‡ Department of Chemical Biology (CBIO), Helmholtz Centre for Infection Research, 38124 Braunschweig, Germany; § Centre for Biological Threats and Special Pathogens, Biological Toxins (ZBS3), Robert Koch Institute, 13353 Berlin, Germany; ∥ German Center for Infection Research (DZIF), Site Hannover-Braunschweig, 38124 Braunschweig, Germany

**Keywords:** Shiga-toxin-producing Escherichia coli detection, Shiga toxin, *N*-glycosidase, sarcin ricin loop, FRET, ssDNA SRL substrates

## Abstract

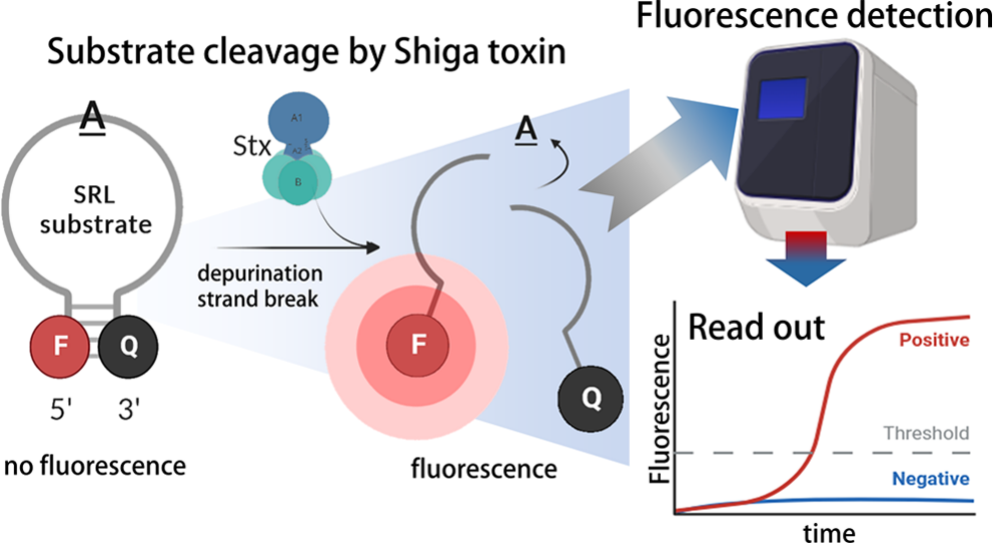

Shiga-toxin-producing *Escherichia coli* (STEC) are important human pathogens causing diarrhea, hemorrhagic colitis, and severe hemolytic uremic syndrome. Timely detection of the multifaceted STEC is of high importance but is challenging and labor-intensive. An easy-to-perform rapid test would be a tremendous advance. Here, the major STEC virulence factor Shiga toxins (Stx), RNA-*N*-glycosidases targeting the sarcin ricin loop (SRL) of 28S rRNA, was used for detection. We designed synthetic FRET-based ssDNA SRL substrates, which conferred a fluorescence signal after cleavage by Stx. Optimal results using bacterial culture supernatants or single colonies were achieved for substrate **StxSense 4** following 30 to 60 min incubation. Stx1 and Stx2 subtypes, diverse STEC serotypes, and *Shigella* were detected. Within a proof-of-principle study, a total of 94 clinical strains were tested, comprising 65 STEC, 11 *Shigella* strains, and 18 strains of other enteropathogenic bacteria without Stx. In conclusion, the assay offers rapid and facile STEC detection based on a real-time readout for Stx activity. Therefore, it may improve STEC risk evaluation, therapy decisions, outbreak, and source detection and simplify research for antimicrobials.

The species *Escherichia coli* is on the one hand part of the commensal intestinal microbiome, and comprises on the other hand pathovars causing disease, such as Shiga-toxin-producing *E. coli* (STEC). STEC may cause a range of symptoms including diarrhea, bloody diarrhea, and severe hemolytic uremic syndrome (HUS) affecting predominantly children up to the age of five. STEC are important zoonotic pathogens and are found in association with animals, such as ruminants, and food, especially meat and milk, but also plant-derived products.^1^ They cause high socioeconomic and economic costs due to their ability to generate large outbreaks, such as the STEC O104:H4 outbreak in 2011 encompassing ∼3000 cases of diarrhea, more than 800 cases of HUS, and 54 fatalities.^2^

STEC possess a variety of virulence factors but Shiga toxins (Stx), AB5 toxins showing RNA-*N-*glycosidase activity, are the most significant virulence factor and are found in all of the diverse STEC.^3^ STEC Stx divides into two major groups, Stx1 and Stx2, which share ∼50% protein sequence identity (Table S1). Stx1 is more closely related to Stx of *Shigella dysenteriae* than Stx2. So far, Stx1 subtypes Stx1a, Stx1c, and Stx1d and Stx2 subtypes Stx2a-o are known in STEC (Table S1).^4,5^ Stx2 and the subtypes Stx2a, Stx2c, and Stx2d are most frequently found in strains associated with HUS and severe disease.^6^ For simplicity, we use the term Stx throughout the article representative for both Stx groups and the various subtypes.

In AB5 toxins, the five B subunits bind to specific receptors, and the A subunit confers the enzymatic activity. The action of Stx in the eukaryotic cell is based on the binding of the B subunit to cellular glycolipid Gb3/CD77 receptors as found on different renal cells, subsequent endocytosis, and release of the furin-processed enzymatically active A1 fragment into the host cell (Figure S1).^3^ Fragment A1 cleaves a single adenine from the 28S rRNA (rRNA) located in the sarcin ricin loop (SRL) of the ribosome, a process called depurination, which blocks the binding of elongation factors to the ribosome, halts protein biosynthesis and thus causes cell death.^3,7^ A similar mechanism is used by related AB type RNA-*N-*glycosidase plant toxins, overall designated ribosome-inactivating proteins (RIPs), such as ricin from *Ricinus communis* and abrin from *Abrus precatorius*. However, those toxins engage other cellular receptors, especially with different oligosaccharide residues like *N*-acetylglucosamine- and galactose-containing receptors.^8^

To facilitate detection of RIP activity, including those of Stx, SRL and shorter RNA or single-stranded DNA (ssDNA) SRL mimics were previously generated. Although *N*-glycosidase activity is, in general, associated with RIPs, their effect on those might be different. For example, ricin requires the recognition sequence GAGA as the minimal sequence for activity in rRNA^9^ but saporin also depurinates adenine-containing 39-mer ssDNA substrates without the recognition sequence.^10^ In addition, Stx releases adenine from 10-mer rRNA SRL substrates including GAGA;^11^ however, it is not known whether short ssDNA SRL can serve as a substrate.

More than 10 years after the large STEC O104:H4 outbreak, timely and qualified detection of STEC including isolate recovery in patients, animals, and food remains of high importance but is still challenging, time-consuming, and labor-intensive. Currently, detection of STEC is increasingly performed by means of molecular tools, such as Stx gene (*stx*) polymerase chain reaction (PCR), from stool or food matrices containing background flora, i.e., from strain mixtures. However, in these instances, isolate recovery is often not successful.^12^ Here, false-positive results may play a role because the PCR signal may be derived from nonvital bacteria or the free *stx* phages.^13^ Correlation of *stx* presence to a specific strain and accordingly isolate recovery is important for the following reasons: for strain risk profiling allowing disease outcome prediction, treatment considerations, decisions on quarantine regulations, or when detected in food for product withdrawal and for subsequent molecular epidemiological analysis permitting disease cluster and respective source recognition.^14^

To obtain an isolate after an enrichment culture and single colony plating from stool or food matrices, usually dozens of colonies need to be analyzed. In addition to *stx* PCR, labor-intense Stx immuno- or toxicity-detection methods are used.^15^ Furthermore, specific resistance phenotypes, such as tellurite resistance,^16^ or metabolic specifics of STEC serotype O157:H7, i.e., lacking sorbitol fermentation and glucuronidase activity, are employed for agar-based detection of STEC single colonies.^17^ However, only a subset of STEC possess these characteristics^16^ and therefore these methods cannot be universally applied.

The so far published activity-based tests for *N-*glycosidase toxins include technically demanding, time-consuming, and cost-intense equipment or procedures, such as LC-MS/MS or multistep enzymatic detection of the released adenine.^11,18−21^ Consequently, the availability of an easy-to-perform and cost-effective rapid test for identification of Stx and STEC on the isolate level would be of tremendous value for infection and pathogen diagnostics. In the study presented here, we developed a fluorescent enzyme substrate for rapid and simplified functional detection of Stx and STEC.

## Results

### Principle of the Enzymatic STEC Detection Assay Based on Stx *N-*Glycosidase Activity

**Figure 1:**
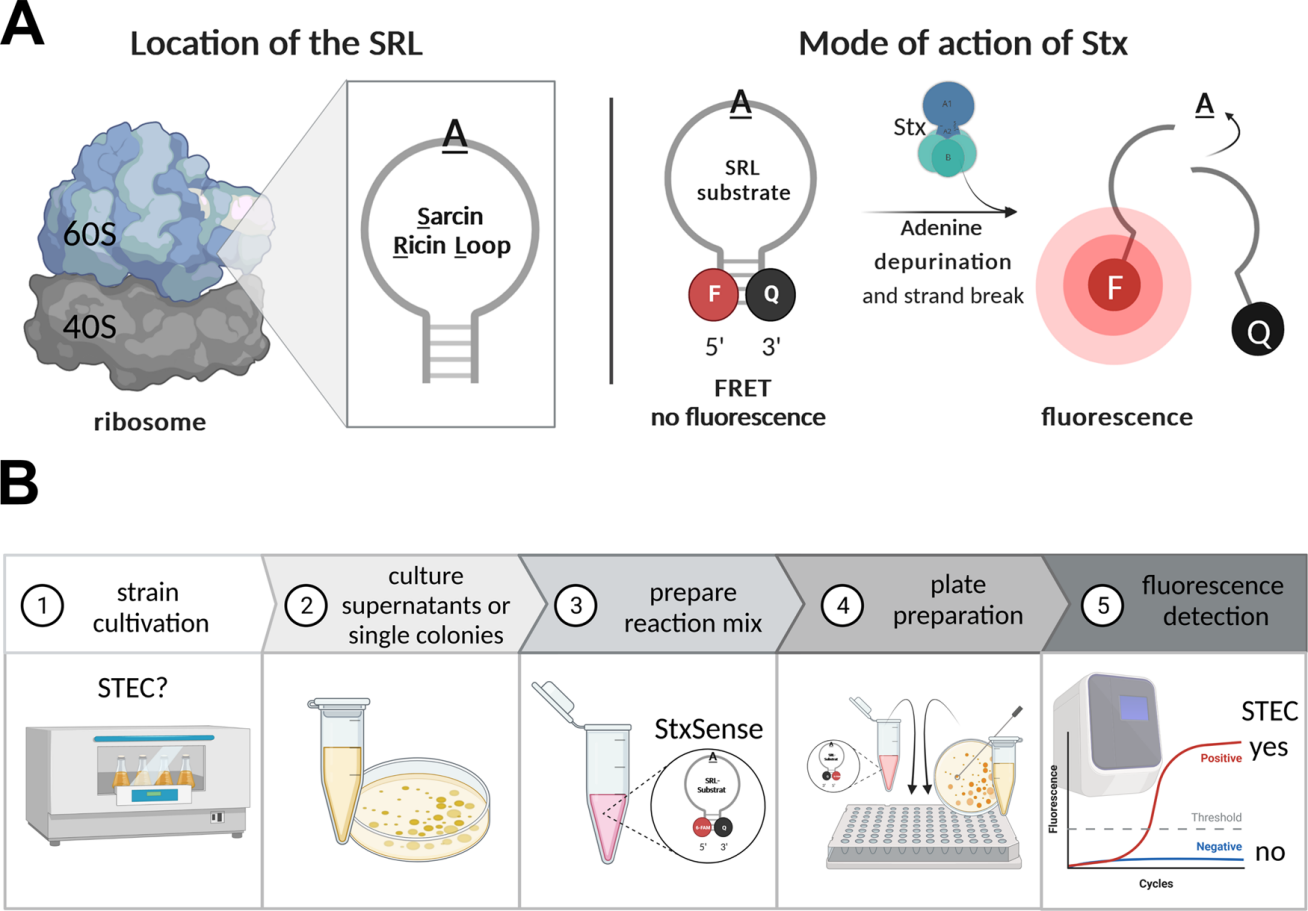
Principle of the enzymatic assay for STEC detection based on Stx *N*-glycosidase activity. (A) Location of the sarcin ricin loop (SRL) targeted by Stx activity within the 60S subunit of the ribosomes. A synthetic SRL mimic with fluorophore (F) and quencher (Q) is used *in vitro* to detect the enzymatic activity of Stx. In the presence of Stx, the SRL adenine is depurinated and the sugar phosphate backbone is cleaved. As a result, the fluorophore and quencher are no longer in physical closeness resulting in a fluorescent signal. (B) Main steps of STEC detection assay.

We aimed to detect Stx *N-*glycosidase activity by employing synthetic SRL mimics equipped with a fluorophore (e.g., 6-Carboxyfluorescein, 6-FAM) and quencher (Q) pair which, when in physical proximity, result in Förster Resonance Energy Transfer (FRET)-based quenching, i.e., no fluorescence (Figure 1A).^22^ In the presence of Stx, SRL is depurinated, resulting in cleavage of the sugar phosphate backbone. When the fluorophore and the quencher are attached to positions down- and upstream of the depurinated site, the loss in physical proximity upon cleavage induces a rapid increase of a fluorescent signal indicative of Stx (Figure 1A). The main steps of the envisioned STEC detection assay include (1) strain cultivation, (2) generation of bacterial supernatants or single colonies, (3) preparation of the reaction mix, (4) reaction plate preparation and reaction start, and (5) fluorescence detection, as depicted in Figure 1B.

### Design of Fluorescently Labeled ssDNA Stx Substrates Based on the SRL

**Figure 2:**
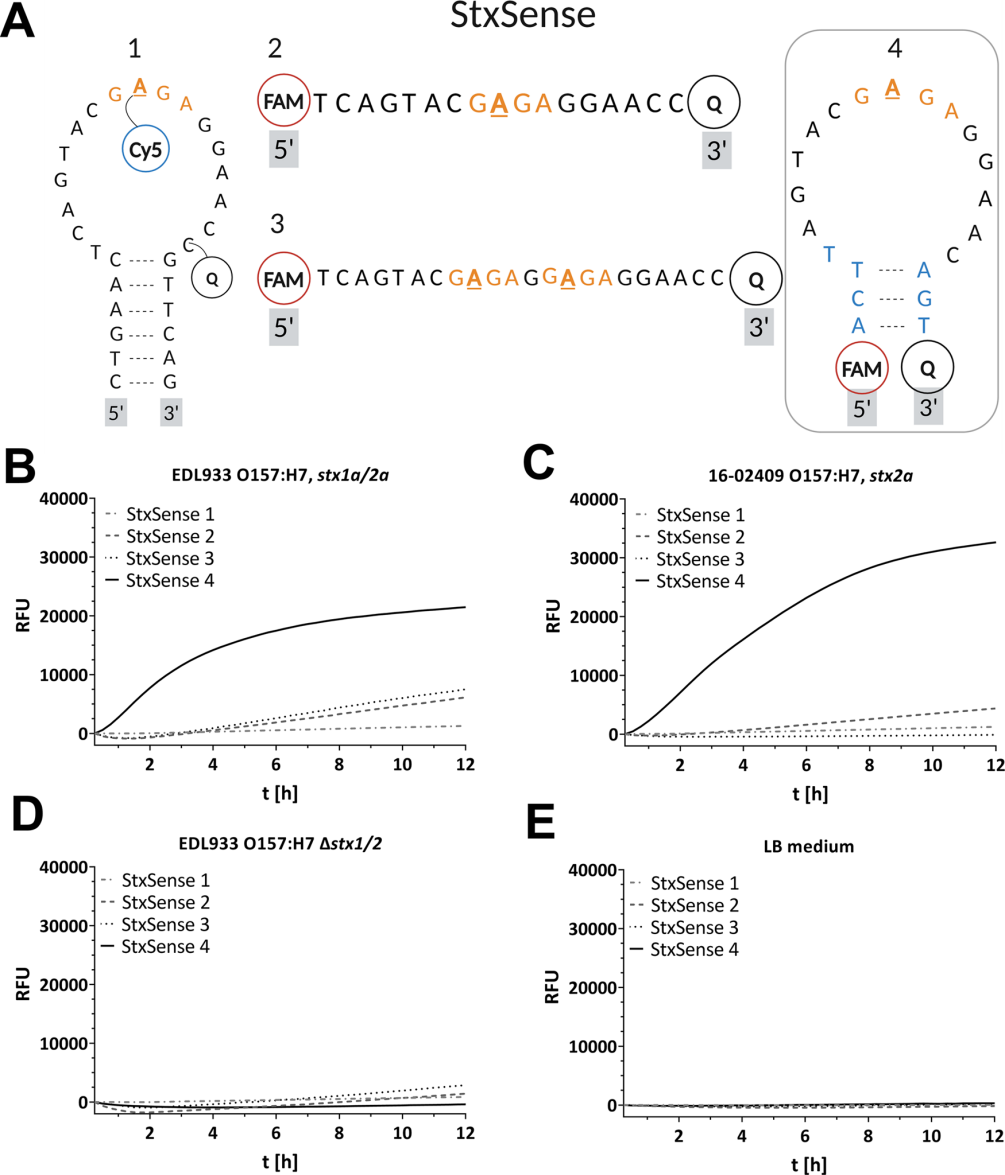
Fluorescently labeled ssDNA substrates based on the SRL detect Stx. (A) Different synthetic SRL substrates StxSense 1 to StxSense 4. Cy5 and FAM (here 6-FAM) denote fluorescence markers and Q a quencher (BMN-Q620 in StxSense 1 and BHQ-1 in StxSense 2–4). Stx recognition sequence is highlighted in orange embedded within the SRL sequence of *R. norvegicus* and the target adenine for depurination is underlined. StxSense3 contains two recognition sequences. StxSense4 contains adapted sequence ends (5′ ACTT and 3′ AGT) compared to the wildtype sequence of *R. norvegicus* to improve the fluorescence signal. (B)–(E) Detected fluorescence as a marker of substrate hydrolysis by Stx from positive control EDL933 O157:H7, test strain STEC 16-02409 O157:H7, and negative controls EDL933 O157:H7 Δ*stx1/2* and LB medium. For both Stx-producing strains (EDL933 O157:H7 and 16-02409 O157:H7), substrate StxSense4 was the optimal substrate for detection of Stx activity. The results represent the medians of triplicate samples (*n* = 3) and are representative of three independent experiments. Statistical analysis was performed by unpaired, double-sided *t* test (*, *p* < 0.05; **, *p* < 0.01; ***, *p* < 0.001), with results compared to those of the EDL933 O157:H7 Δ*stx1/2*. For statistics and Vero cell cytotoxicity assay see Figure S6. RFU, relative fluorescence units; *t* [h], time [hours].

In order to increase the stability of the reagent, an ssDNA template was chosen instead of the natural Stx RNA-based substrate. We designed four ssDNA substrates StxSense 1 to StxSense 4 of different lengths (17 to 29 nt) based on the SRL sequence from *Rattus norvegicus* (Figure 2A, Table 1). StxSense 1, 2, and 4 included one recognition sequence GAGA (target adenine for depurination underlined), while StxSense 3 contained two of these to promote the chance of cleavage.^23^ Cyanine5 (Cy5) and 6-FAM were used as fluorescence markers; the first was coupled to the targeted adenine in StxSense 1 and the latter was located at the 5′ end of the substrates in StxSense 2 to StxSense 4. Quencher BMN-Q620 was used in StxSense 1 and was located within the substrate sequence, whereas quencher BHQ-1 was located at the 3′ end in substrates StxSense 2 to 4. Further, StxSense 4 comprised adapted sequence ends (5′ ACTT and 3′ AGT) to potentially improve the fluorescence signal after cleavage. Finally, the base pairing of the two most distal bases brings the fluorophore and quencher closer together, so that the quenching effect would be optimal (Figure 2A).

**Table 1:** Synthetic ssDNA Substrates for the Detection of Stx *N*-Glycosidase Activity^a^

substrate	sequence (5′→ 3′)	k-mer	fluorophore (F)	quencher (Q)	characteristics
StxSense 1	CTG AAC TCA GTA CGA (Cy5) GAG GAA CC GTT CAG (Q)	29	Cy5	BMN-Q620	adenine labeling
StxSense 2	(FAM) TCA GTA CGA GAG GAA CC (Q)	17	6-FAM (5′ end)	BHQ-1 (3′ end)	reduced SRL sequence compared to StxSense 1
Stxsense 3	(FAM) TCA GTA CGA GAG GAG AGG AAC C (Q)	21			double recognition sequence GAGA
StxSense 4	(FAM) AC TTA GTA CGA GAG GA ACAG T (Q)	21			modified ends before F/Q

a Studied SRL substrates and their respective sequences, features, and fluorophores (F): Cy5 = Cy5 fluorophore; FAM = 6-FAM fluorophore (fluorescein), and quencher (Q). Recognition sequence (GAGA) including the adenine (A) targeted for depurination.

### Substrate StxSense 4 Showed Highest Stx-Dependent Fluorescence Using STEC Culture Supernatants

To test the designed substrates for Stx detection, culture supernatants of two STEC strains, specifically, reference strain STEC O157:H7 EDL933 comprising *stx1a* and *stx2a* and clinical STEC isolate 16-02409 comprising *stx2a* were incubated with 2 μM substrates StxSense 1 to StxSense 4 in depurination buffer at pH 4 and at 44 °C for up to 12 h. The fluorescence readouts were compared to the *stx1/2*-deficient EDL933 Δ*stx1/2* strain and LB medium, which served as negative controls. For both STEC strains, substrate StxSense 4 induced the highest fluorescence readout among the four substrates from ∼30 min to 1 h (Figure 2B,C, statistics Figure S2A,B), whereas the negative controls did not show substantial fluorescence over the whole time period for StxSense 4 (Figure 2D,E, statistics Figure S2C,D). Further, we tested other intestinal *E. coli* pathovars and other intestinal pathogens without *stx* for assay interference. EPEC, EAEC, and EIEC strains (Figure S2E, statistics Figure S2F) and additional strains of different *Salmonella enterica* serovars and of *Yersinia enterocolitica* (Figure S2G, statistics Figure S2H) did not show cross-reactivity. Additionally, the culture supernatants of the tested strains behaved as expected in Vero cell cytotoxicity assays, which is an established method for Stx detection. Specifically, only strains comprising *stx*, such as STEC strain EDL933, showed reduced cell viability, but not EDL Δ*stx1/2* or other intestinal pathogenic bacteria all without *stx* (Figure S2I,J).

Other RIPs, such as ricin, show specificity for the ribosomal recognition sequence GAGA, which is essential for activity (9). To analyze whether this motif is important for the assay described here, StxSense 4 substrates with an altered GAGA recognition sequence, such as GGGG, GGGA, and GAGG, were incubated with STEC culture supernatants of the two positive control strains. In both occasions, the overall highest fluorescence signal was obtained for StxSense 4 with unchanged GAGA recognition sequence (Figure S2K,L). The negative control (EDL933 Δ*stx1/2* culture supernatant) did not yield substantial fluorescent readout (Figure S2M). In addition, cleavage of StxSense 4 was found by STEC culture supernatant (strains EDL933 and 16-02409) but not by negative controls (EDL933 Δ*stx1/2* or LB) which is a precondition for fluorescence development (Figure S2N). In conclusion, the experiments indicated that substrate StxSense 4 containing the GAGA motif was most suited for STEC and Stx detection and that other Stx-negative intestinal pathogenic bacteria did not show cross-reactivity. Therefore, StxSense 4 was used for all of the further experiments in the study.

### Optimal Assay Conditions for Stx Detection in Culture Supernatants

We optimized important assay parameters such as substrate concentration, concentration of the depurination buffer ammonium acetate, the nature of the 96-well plates, and reaction temperature. First, the StxSense 4 concentration was analyzed in the range of 1 to 8 μM. For robust Stx detection from STEC EDL933 culture supernatants within 30 min/1 h, 2 μM StxSense 4 was sufficient (Figure S3A and statistics Figure S3B). Importantly, fluorescence readout at earlier time points further increased especially to a concentration of 5 μM. Second, the depurination buffer ammonium acetate (pH 4) was applied in 10 and 100 mM concentrations. A 100 mM ammonium acetate buffer led to higher readouts for STEC strains EDL933 and 16-02409 (Figure S3C and statistics Figure S3D). Third, the nature of the 96-well plates had an influence on Stx detection. For both STEC culture supernatants, the white plates performed better than the transparent ones (Figure S3E and statistics Figure S3F). Fourth, the influence of the reaction temperature was analyzed in the range of 37 to 45 °C. For STEC EDL933 culture supernatants, the fluorescence readout was highest from ∼44 °C, but substantial readouts were already observed from 37 °C (Figure S3G and statistics Figure S3H). To account for both maximal signal as well as cost-effectiveness, the following assay parameters were defined for STEC culture supernatants and used throughout the study: 2 μM StxSense 4, 100 mM ammonium acetate, white 96-well plates, and reaction temperature of 44 °C.

### The Limit of Detection (LOD) for the Assay Lies in the Range of 10–29 ng/mL

**Figure 3:**
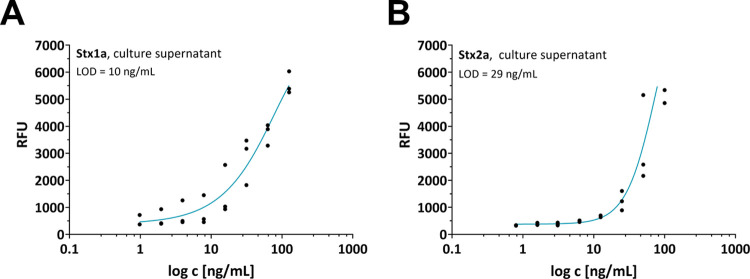
Limit of detection correlation analysis for STEC Stx1a and Stx2a. Concentration-dependent analysis of Stx activity in 100 mM ammonium acetate, pH 4, 44 °C of (A) strains 20-01044 O111:H8, *stx1a* and (B) 16-02409 O157:H7, *stx2a*. Plotted are the log c [ng/mL] of the Stx ELISA quantified culture supernatants versus the detected RFU. The correlation was obtained using an exponential function and equation LOD = 3 * SD/b and LOD is limit of detection, SD is the standard deviation and b is the slope of regression curve. Shown is the representative plot of one determination in triplicates from a total of three representative independent determinations (*n* = 3). RFU, Relative Fluorescence Units.

We further analyzed the limit of detection of this assay. To that end, we used culture supernatants of a solely Stx1a- and another solely Stx2a-producing strain, 20-01044 O111:H8 and 16-02409 O157:H7, respectively. The culture supernatants were diluted, and the Stx concentrations were determined by sandwich ELISA; in parallel RFU readout was analyzed by means of the novel assay. LOD for Stx1a was determined as 10 ng/mL (Figure 3A) and LOD for Stx2a was 29 ng/mL (Figure 3B).

### Detection of Stx1 and Stx2 Subtypes across Different STEC Serotypes and *Shigella* spp

**Figure 4:**
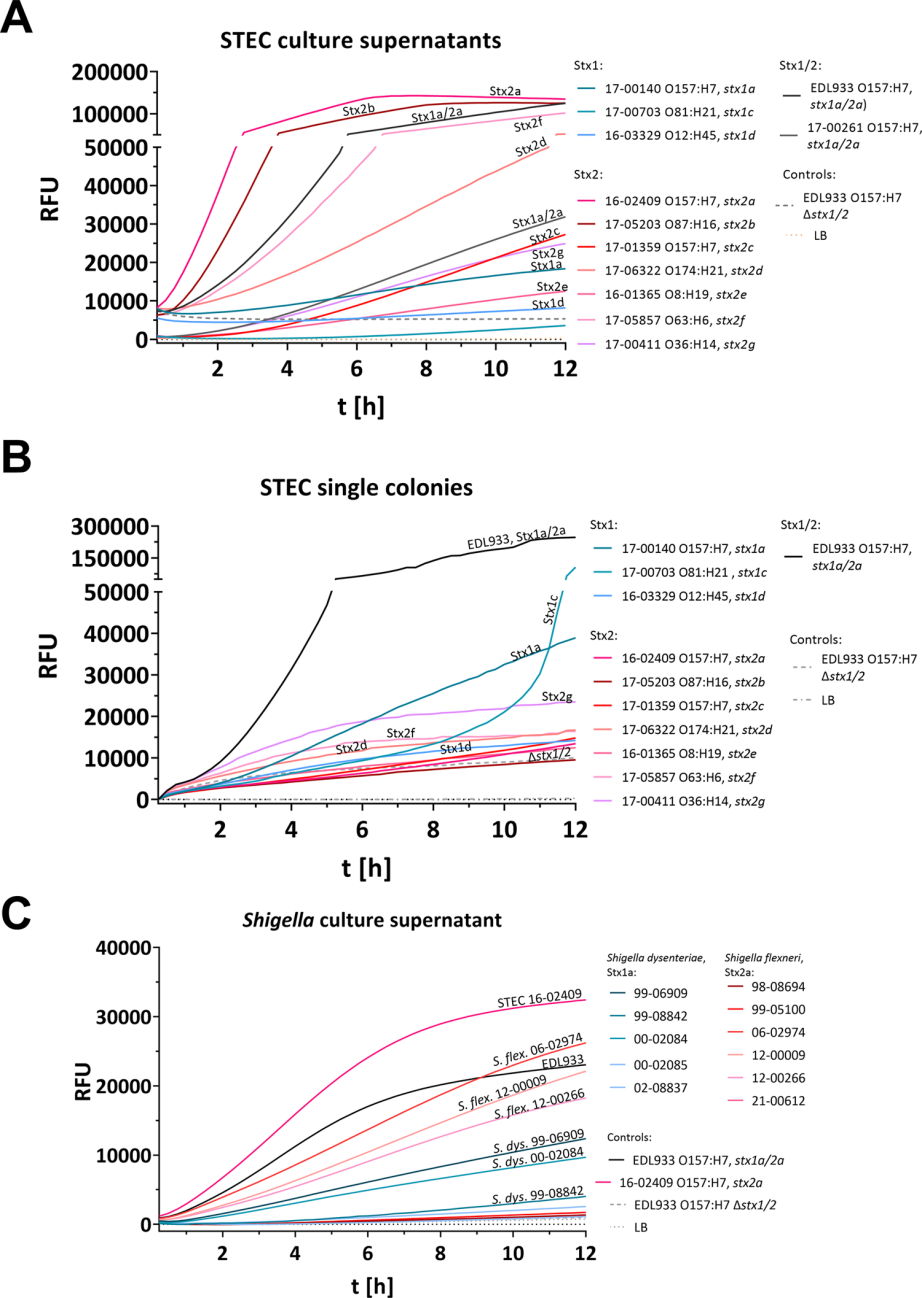
Detection of relevant Stx1 and Stx2 subtypes was possible in a variety of STEC serotypes and *Shigella* spp. Detected fluorescence as a marker of substrate hydrolysis by Stx from STEC culture supernatants and single colonies. (A) The assay detects Stx2 subtypes and some Stx1 subtypes when culture supernatants of bacteria grown in LB medium supplemented with 12 ng/mL ciprofloxacin are used. Since Stx1 is scarcely released into the culture supernatant, detection of Stx1 in culture supernatants is strain-dependent. Associated graphs in the supplement are presented in Figure S4A–C (B) Using single colonies grown on LB agar supplemented with 12 ng/mL ciprofloxacin, Stx1- and Stx2-producing STEC strains are detected within 10 h. (C) *Shigella flexneri* strains releasing Stx into culture supernatants are detected by the assay. The results represent the medians of triplicate samples (*n* = 3) and are representative of three independent experiments. Statistical analysis was performed by Mann–Whitney test (A, B) for non-normally distributed samples and unpaired, double-sided *t* test (C) for normally distributed samples (*, *p* < 0.05; **, *p* < 0.01; ***, *p* < 0.001), with results compared to those of the EDL933 O157:H7 Δ*stx*1/2 (negative control; -- [gray dashed lines]). For statistics and Vero cell cytotoxicity assay see Figures S8 and S9. RFU, relative fluorescence units; *t* [h], time [hours].

Three different subtypes of *stx1* and 14 subtypes of *stx2* have been described.^4,5^ Therefore, we analyzed whether the most relevant Stx subtypes are detected by the assay. To this end, additional STEC strains of serotypes harboring *stx1a, stx1c, stx1d* or/and *stx2a* to *stx2g* were analyzed using culture supernatants or single colonies. The latter was additionally implemented because the release of Stx1 into culture supernatants is lower compared to Stx2.^24^ In culture supernatants, strains showing Stx2a, Stx2b, a combination of Stx1a and Stx2a, Stx2f, Stx2g, Stx2d, and Stx2c were detected between 30 min and 2 h reaction time, whereas the ones with other Stx types, including Stx2e, and Stx1a, required more time (Figure 4A,B, statistics Figure S4A,B). A weak signal was observed for Stx1c and Stx1d after >10 h (Figure 4A, statistics Figure S4A,B). All tested *stx*-positive strains revealed Vero cell toxicity as a standard measure of Stx (Figure S4C).

Using single colonies of the same strains instead of culture supernatants, especially the combination of Stx1a and Stx2a, several Stx2 (Stx2g, Stx2f, Stx2d), and after ca. 3–4h Stx1a, Stx1c, Stx1d and some other Stx2 were detected, but also the background of strain EDL933 Δ*stx1/2* rose (Figure 4B, statistics Figure S4D). When the assay was performed with single colonies, we noted that 10 mM ammonium acetate buffer instead of 100 mM buffer produced higher fluorescence signals and therefore 10 mM buffer was used for single colony analysis (Figure S4E and statistics Figure S4F).

Various clinical STEC strains of frequently disease-associated O types in addition to O157, such as O26, O91, O111, O113, O121, and O145 all resulted in a positive Stx assay signal from culture supernatants (Figure S4G, statistics Figure S4H, and Vero cell assay Figure S4I). Further, 46 additional clinical STEC strains from the NRC collection of a variety of serotypes and *stx* subtypes were tested based on culture supernatants and were all detected by the Stx enzymatic assay, except six strains which did not result in a significant RFU change (five strains showing *stx2e* and one *stx2d*) and correspondingly did not reveal cytotoxicity (Figure S4J,L,N; statistics Figure S4K,M,O, cytotoxicity Figure S4P).

Further, some *Shigella* strains may harbor *stx*.^1^ Indeed, *Shigella flexneri* strains releasing Stx2*a* into the culture supernatant, such as strains 06-02974, 12-00009, and 12-00266, or *S. dysenteriae* showing Stx1a, such as strains 99-06909, and 99-08842, 00-02084, resulted in a fluorescence signal, conferred cytotoxicity toward Vero cells, and resulted in Stx detection by Stx ELISA (statistics Figure S5A,B, cytotoxicity Figure S5C, and Stx ELISA Figure S5D). As expected, the five *Shigella* strains which did not result in a fluorescent signal, did not show cytotoxicity toward Vero cells and three of the six did not release Stx into the culture supernatant (Figure S5A–D).

In conclusion, standard assay conditions allowed for the detection of all 59 STEC strains producing Vero cell cytotoxicity, comprising the manifold Stx1 and Stx2 subtypes and a wide variety of different STEC serotypes and also *Shigella* spp. Further, implementation of culture supernatants is optimal for Stx2-producing strains, and single colony analysis can improve the detection of Stx1-producing strains.

## Discussion

Timely and qualified detection of STEC including isolate recovery is of high importance but is still challenging and labor-intense. Current PCR procedures target *stx*, but the isolation of STEC from the PCR-positive samples succeeds in only about 42% of cases.^12^ Although PCR methods for the determination of *stx* subtypes correlating to disease severity are available,^25^ they do not allow conclusions on whether active Stx is produced and on Stx amounts; two important parameters which might be linked to the fate of disease and progression into HUS. In addition, other tests targeting resistance or metabolic features are only covering a subset of the multifaceted STEC group. Thus, an easy-to-perform rapid test for all STEC values represents an unmet diagnostic need. Therefore, we developed an STEC detection method based on the *N-*glycosidase activity of Stx.

To that means, we designed different synthetic FRET substrates mimicking the SRL which after cleavage by Stx result in a real-time fluorescence signal (Figures 1A and 2A). Substrate StxSense 4 yielded in the highest fluorescent readout (Figure 2B,C). The other substrates either did not respond to Stx at all, such as StxSense 1, containing the fluorescence marker at the target adenine for depurination, or revealed a fluorescence signal significantly lower than that of StxSense 4. These, StxSense 2 and StxSense 3, like the optimal substrate StxSense 4, had the fluorescence marker at the 5′ end and the quencher on the 3′ end. Differences compared to StxSense 4 were that in StxSense 2 and StxSense3 no modified ends were added. In StxSense 3, an additional GAGA recognition sequence was added which however did not lead to improved activity. Therefore, we conclude substrate StxSense 4 was optimal, which contained a central recognition site GAGA, important for the assay, the fluorescence marker, and quencher at the distal ends of the substrate flanked by modified ends.

Previous work on RIP substrates, including Stx substrates, were not linked to a fluorescence marker and encompassed typically RNA and for plant RIPs in some cases ssDNA.^18^ The sequences mostly included the GAGA recognition site and varied in length between 10 and 29 bases.^18,20^ Often, the release of adenine was quantified by means of enzymatic ADP to ATP conversion or liquid chromatography coupled mass spectrometry (LC-MS).

In a 2013 patent (No.: US 10,907,193 B2), a fluorescence-labeled ssDNA substrate was used to detect the activity of ricin in a real-time approach. Ricin depurinates the specific adenine of the recognition sequence in a synthetic ssDNA substrate and creates an abasic site. By adding a lyase, the DNA is cleaved at this site, and accordingly a fluorescence signal is generated. It seems that the strand break might be a limiting factor for some plant RIPs requiring the addition of strand-breaking enzymes. This is not the case for Stx detection, as shown in our study. The substrates used in the patent varied in length from 26 to 31 nt and contained the fluorescent group at the 5′ and the quencher at the 3′ end. It was concluded that both shape and sequence of the substrates, some of which did not harbor the GAGA recognition sequence because certain plant toxins might depurinate any adenine in the sequence,^10^ seem to play a role in catalysis by ricin.

**Figure 5:**
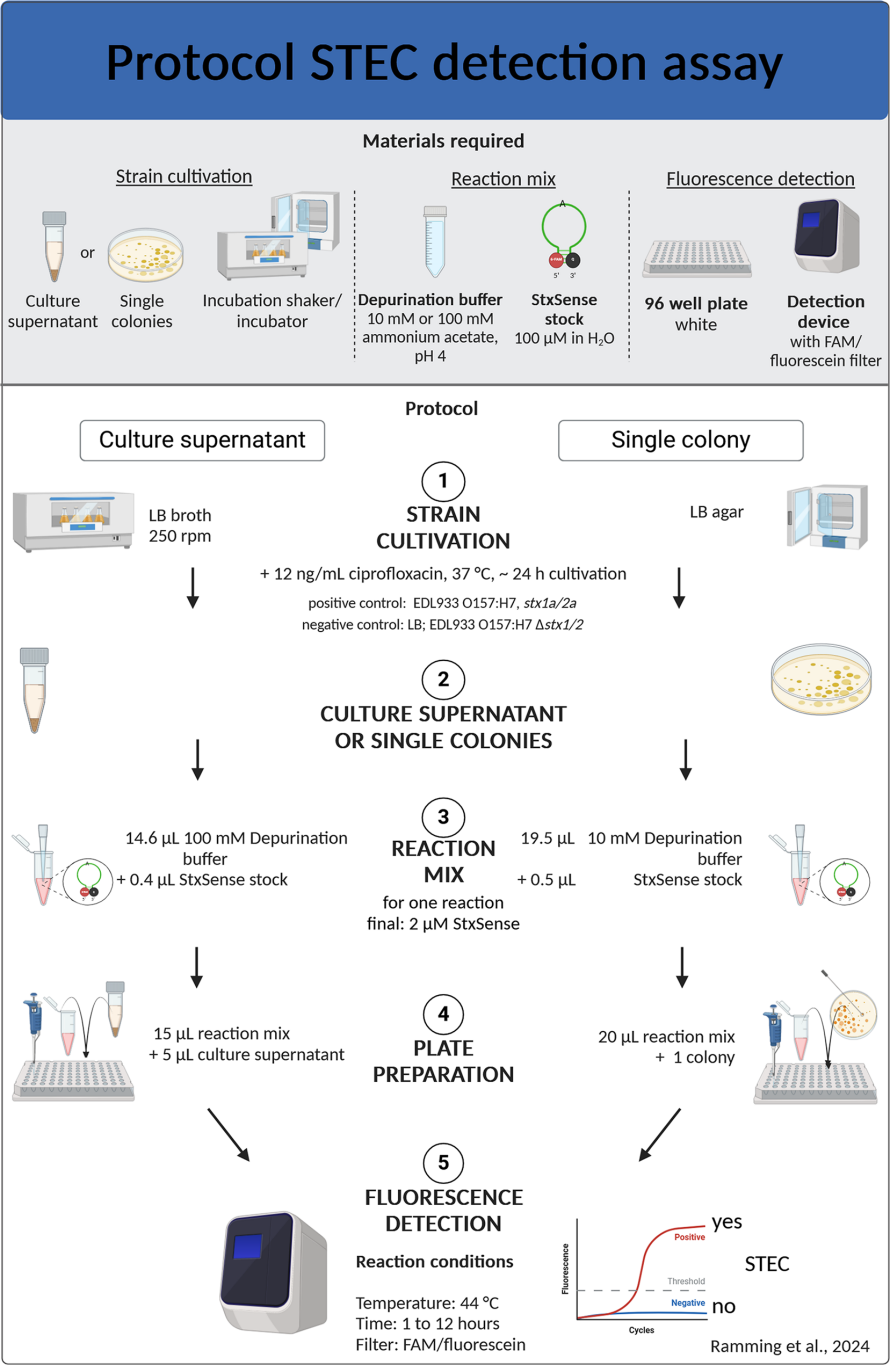
Assay protocol for the STEC detection assay. Overview of the materials required and the steps involved in the enzymatic assay for Shiga toxin detection from culture supernatants or single colonies. Briefly, after standard cultivation of STEC in LB broth or on LB agar, both with 12 ng/mL ciprofloxacin, the culture supernatant or single colonies are added to the reaction mix (ammonium acetate buffer pH 4 containing SRL substrate) in a well of a 96-well plate. The enzymatic reaction is performed at 44 °C for up to 12 h and the fluorescent signal (RFU, Relative Fluorescent Unit) is read with a detection device containing the appropriate FAM/fluorescein filter, such as a real-time cycler or fluorescence gel imager. The supernatant or single colonies of EDL933 O157:H7, *stx1a/2a* are used as a positive control; LB, *E. coli* C600, or EDL933 O157:H7 Δ*stx1/*2 as negative controls defining the threshold.

The assay introduced here requires minimal effort (Figures 1B and 5) and is as cost-effective as PCR methods. Specifically, bacterial culture supernatants or single colonies are directly incubated with reaction buffer composed of the pH 4 depurination buffer and the StxSense 4 enzyme substrate. The novel assay importantly only requires the fluorescent SRL substrate which can be easily obtained from a commercial oligo/probe synthesizing company. However, for PCR approaches primers, dNTPs, and a polymerase and for qPCR, additional DNA-binding dyes or a fluorescent probe are necessary. In this proof-of-principle study, a real-time PCR instrument was used to detect the fluorescence signal; however, it may be possible to use a microplate reader with a suitable filter instead. In such cases, it should be considered that the required reaction volume, and thus the sample volume, is higher than when using PCR plates.

Li and Tumer^11^ used 2 μM substrate concentration for the detection of Stx activity. Here, Stx activity was determined by depurination of a 10-mer RNA substrate, further enzymatic conversion of the released adenine into ATP, and detection of a luminescent readout. A minimal substrate concentration of 2 μM was also found in our experiments for Stx detection, and fluorescence intensity increased for the tested reference strains up to a substrate concentration of 5 μM (Figure S3A). For example, when 2 μM StxSense 4 substrate was used, costs are ∼0.20 or ∼0.50 Euro/sample for 5 μM substrate concentration, respectively. Other materials required for the depurination buffer and 96-well plates sum up to less than 0.10 Euro/sample. Therefore, costs for the here-described assay are in the range or even below PCR and qPCR.^26^ Furthermore, the LOD lies in the range of ∼10 to ∼29 ng/mL (Figure 3A,B) and is therefore higher but still in the dimension of the published ranges for established methods, such as ELISA and the Vero cell cytotoxicity test.^27−31^ In conclusion, the combination of simplicity, cost-effectiveness, and sensitivity is a clear advantage of the novel FRET-based assay.

Our data show that higher temperatures, for example, 44 °C, were optimal for the assay readout. This observation has been described before for Stx and other RIPs, and even strand breaks after substrate depurination by plant toxins are further promoted by final short-term incubations at 90 °C.^23^ Additionally, acidic pH and a combination with higher temperatures is also beneficial for Stx enzymatic assays.^32^ This may be due to structural reorganizations occurring in Stx which open the catalytic site for better substrate access.^11^ Nevertheless, it is possible to perform the assay at 37 °C without a major reduction in fluorescence intensity (Figure S3G,H). This fact might be important for a possible future adaptation of the assay as an agar plate-based STEC growth and detection medium.

In our study, we analyzed a total of 94 different strains, including 65 STEC and 11 *Shigella* strains. All strains producing functional Stx in the detection sample yielded in Stx activity in the novel assay. Therefore, the assay can promote analysis of STEC and in addition may allow faster detection of relevant STEC strains, particularly such strains producing cytotoxicity and such predominantly associated with HUS development. Indeed, our data highlight high and consistent fluorescence readouts for Stx subtypes Stx2a, Stx2c, and Stx2d known for their superior HUS association.^33^ Interestingly, also STEC producing Stx2b showed high fluorescence readouts which may correlate to recent EFSA data about prolonged course of disease for such infections.^34^

In addition to facilitating STEC detection, our assay provides an alternative method for the analysis of Stx enzyme activity. So far, methods including (1) Stx toxicity toward susceptible eukaryotic cells, especially Vero cells, (2) ribosome inactivation in a cell-free *E. coli* protein synthesis system, (3) eukaryotic test systems detecting ribosome inactivation by Stx, or (4) depurination of SRL mimics without fluorescence marker, as outlined above, are used. Other elegant current assays for RIP detection include a luminescence assay^20^ or a qRT-PCR.^21^ In the first one, the adenine cleaved by the RIP from 60S yeast ribosomes and stem-loop RNA is converted to ATP in a downstream reaction with ATPlite. ATP is then used by luciferase enzymatic reaction, which produces a light readout. In the second method, SRL is depurinated by RIP leading to an abasic site. A reverse transcriptase is used to fill the abasic site with an adenine. In combination with specific primers that detect the A-T conversion in the cDNA, the catalytic activity of RIP is determined.

The methods mostly require specialized equipment, such as mass spectrometers, eukaryotic cell culture, expensive reagents, or ribosome or sensitive DNA/RNA preparations.^20^ All of these approaches are more complex and laborious than the method introduced here. Furthermore, some rely on multistep reaction-based detection, whereas the here-introduced assay encompasses a one-step reaction. The new method therefore might open up alternative test strategies for novel therapeutic approaches. Currently, there is no effective treatment for HUS, and only supportive care, such as fluid volume management, is recommended.^35^ Applying the novel Stx enzyme assay, antimicrobials can now be easily tested for their potential of Stx phage induction and accordingly therapy-associated increased Stx production.^36^ Further, the novel method may facilitate screens for substances that inhibit Stx activity.

## Materials and Methods

### Experimental Design

In the study presented here, we developed a fluorescent enzyme substrate for rapid and simplified functional detection of Stx and STEC. We designed synthetic FRET-based ssDNA SRL substrates that conferred a fluorescence signal after cleavage by Stx. We further aimed to optimize the reaction conditions and in total validated the assay for 65 STEC strains and 11 *Shigella* strains, and 17 strains not producing Stx. Most of the strains originated from the collection of the German National Reference Centre (NRC) for *Salmonella* and other bacterial enteric pathogens of the Robert Koch Institute.

### Bacterial Strains, Eukaryotic Cell Lines, Growth Conditions, and Preparation of Culture Supernatants, and Single Colonies

Strains used in the study are listed in Table S2 (STEC), Table S3 (other *E. coli* pathovars without *stx*), Table S4 (other enteropathogenic bacteria without *stx*), and Table S5 (*Shigella*). A variety of strains were employed: 65 STEC strains harboring *stx1* and/or *stx2* including different *stx* subtypes (s*tx1a, stx1c, stx1d, stx2a-g*) and 30 different serotypes were analyzed (Table S2). Reference strains of STEC O157:H7 EDL933 harboring *stx1a* and *stx2a* and an isogenic *stx*-negative knockout mutant EDL933 Δ*stx1/2* were used as controls (Table S2). Further strains, such as other intestinal pathogenic *E. coli* belonging to pathovars EPEC, EAEC, EIEC (Table S3), and other intestinal pathogenic bacteria, such as *S. enterica*, *Y. enterocolitica*, all without *stx* (Table S4), and *Shigella* spp. with *stx1*a or *stx2a* were used (Table S5). All strains, excluding the reference strains, are clinical strains and were collected and characterized by NRC for *Salmonella* and other Bacterial Enteric Pathogens.

All bacterial strains were streaked out and grown on LB agar overnight for single colonies at 37 °C or cultivated in LB broth (high NaCl 10 g/L) for 18 h at 250 rpm and 37 °C. 12 ng/mL ciprofloxacin (Cip, Sigma-Aldrich, Darmstadt, Germany) was used in these media as an alternative for mitomycin C (MMC) for the induction of the Stx production.^36^ Culture supernatants were obtained by centrifugation (8000*g*, 10 min) and filtration (0.22 μm filters; Sartorius, Göttingen, Germany). Stx production was tested using the Vero cell cytotoxicity assay (supplement), Enzyme Linked Immunosorbent Assay (ELISA) (supplement), and the here-described Stx activity assay. Culture supernatants were stored at 4 °C for up to 1 week or at −20 °C for up to six months.

The Vero cell line (ACC33; German Collection of Microorganisms and Cell Cultures GmbH, Braunschweig, Germany) was cultured at 37 °C, 5% CO_2_ in DMEM (Capricorn Scientific, Ebsdorfergrund, Germany) with 10% fetal bovine serum (FBS; Capricorn Scientific).

### *E. coli* Serotyping and Virulence Gene Analysis

The presence of *stx* was determined using colony PCR after growth on LB agar as described by Cebula et al.^37^ Stx subtype and the presence of *eaeA* were determined by PCR or were extracted from the genome sequence.^4,38,39^*E. coli* O and H antigens were determined by microtiter agglutination method or extraction from the genome sequence as described elsewhere.^39,40^

### Enzyme Linked Immunosorbent Assay (ELISA)

The qualitative analysis of Stx in culture supernatants was performed using commercially available Ridascreen Verotoxin Stx ELISA (r-Biopharm AG; Pfungstadt, Germany) as specified by the manufacturer. For the quantitative analysis, a custom-made Stx sandwich ELISA was used. Briefly, a 96-well plate (MaxiSorp; Nunc, Thermo Fisher Scientific, Germany) was coated with 10 μg/mL of a capture antibody in 50 μL of Phosphate Buffered Saline (PBS) (13C4, hybridoma cell line from American Type Culture Collection, Manassas, for Stx1; MBS311736, MyBioSource Inc., San Diego, for Stx2) overnight at 4 °C and blocked with casein buffer (Senova, Jena, Germany) for 1 h at room temperature. After washing using PBS with 0.1% Tween 20, 50 μL of diluted STEC culture supernatants or Stx standard dilution series were added and incubated for 2 h at room temperature. After washing, the bound Stx of STEC culture supernatants was detected using a biotinylated detection antibody (MBS311734 for Stx1, MyBioSource Inc., San Diego; 11E10, hybridoma cell line from American Type Culture Collection, Manassas; and BB12,^41^ Toxin Technology Inc., Sarasota, for Stx2) incubated for 1 h at room temperature. After washing, the ELISA was developed with PolyHRP40 (Senova GmbH; Jena, Germany) and substrate 3,3′,5,5′-tetramethybenzinine (TMB, SeramunBlau slow2 50, Seramun Diagnostika, Heidesee, Germany). The color reaction was stopped by 0.25 M sulfuric acid. After absorption detection at 450 nm versus 620 nm (Tecan Infinite M200), the concentration of Stx in culture supernatants was calculated using Stx1 and Stx2 standards (Toxin Technology Inc.) of known concentration in the range from 0.3 pg/mL to 100 ng/mL.

### StxSense SRL Substrates for Stx Detection

Four synthetic ssDNA substrates for Stx detection were designed based on the SRL sequence of *R. norvegicus* and commercially synthesized (Integrated DNA Technologies [idt], Leuven, Belgium; or biomers.net GmbH; Ulm, Germany). These substrates include the described SRL recognition sequence GAGA for RIPs and fluorophore/quencher pairs (Table 1). For all substrates, the quencher was at the 3′ end. **StxSense 1** contained a Cy5 fluorophore at the central adenine, which is specifically depurinated by Stx. All other substrates, substrates **StxSense 2–StxSense 4**, were labeled with a 6-FAM fluorophore at the 5′ end. Substrates were synthesized by a commercial provider (e.g., integrated DNA technologies, idt) and quality control data for **StxSense 1–StxSense 4** are shown in Figures S6–S9.

### Stx *N-*Glycosidase Enzyme Assay Using Culture Supernatants and Single Colonies

StxSense synthetic ssDNA substrates that are fluorophore-coupled (Table 1) were used to detect the *N-*glycosidase activity of Stx. For culture supernatant samples, 14.6 μL of 100 mM ammonium acetate (Fluka, item no. 09690, VWR International LLC, Pennsylvania), adjusted to pH 4 with HCl (32%; item no. 4625.1, Carl Roth GmbH, Karlsruhe, Germany) and 0.4 μL of the substrate (100 mM stock in dH_2_O; 2 μM final test concentration) were mixed (reaction mix) and transferred into a white 96-well plate (Eppendorf Twin-Tec, item no. 0030132718, Eppendorf SE; Hamburg, Germany). For single colony samples, 19.5 μL of 10 mM ammonium acetate, adjusted to pH 4 with HCl, and 0.5 μL of the substrate (100 mM in dH_2_O; 2 μM final concentration) were mixed (reaction mix) and transferred into a white 96-well plate. Subsequently, 5 μL of the culture supernatant or a single colony prepared as described above, was added. The assay was carried out using the following parameters for each cycle: isothermic reaction 44 °C, detection of fluorescence, for StxSense 1 Cy5 or StxSense 2 – StxSense 4 FAM filter, after 1 cycle after 15 min reaction time using a real-time instrument (CFX96; Bio-Rad Laboratories, California). In total, 48 cycles were programmed resulting in an overall reaction time of 12 h. Stx activity was analyzed by depicting the Relative Fluorescence Units (RFU) versus time graphically in GraphPad Prism (GraphPad Software; San Diego).

### Specificity of Stx Detection

The Stx enzyme assay was examined by using STEC strains expressing *stx1* and/or *stx2* as well as the *stx* subtypes (*stx1a, c, d*, and *stx2a-g*). In addition to strains of the serotype O157:H7, strains of 29 additional STEC serotypes were analyzed. To exclude cross-reactivity, other intestinal pathogenic *E. coli* (EAEC, EPEC, EIEC) and other intestinal pathogenic bacteria (such as *Yersinia*, *Salmonella*) without *stx* were tested.

### Sensitivity of Stx Detection

The limit of detection (LOD) was calculated using quantified culture supernatants (see the ELISA section). For STEC culture supernatants comprising Stx subtypes Stx1a and Stx2a, a 1:2 dilution series in the range of 1 to 126 ng/mL (Stx1a) or 138 ng/mL (Stx2a), respectively, was prepared and 5 μL of each dilution step were analyzed for enzyme activity. The LOD was determined from regression curves using GraphPad Prism and the equation LOD (RFU) = 3*σ/S, in which LOD is the limit of detection, σ is the standard deviation, and S is the slope of the regression curve.

### Data Analysis

Values of the enzyme assay curves were obtained with CFX Maestro (Bio-Rad Laboratories, Inc.) and were illustrated and analyzed using GraphPad Prism (GraphPad Software; San Diego). Statistical analysis was performed for the RFU values at 2, 4, 8, or/and 12 h. All experiments were examined for a normal distribution. Normally distributed values were statistically analyzed using double-sided *t* test, corrected with Welch, whereas for non-normally distributed values, the Mann–Whitney-*U*-test was used (see the Supporting Information).

### Graphical Representation

Graphs were created using GraphPad Prism, version 9.10.0.22 (GraphPad Software, LLC), and figures were created using BioRender.com (Toronto, Canada).

## Data Availability

The data underlying this article are available in the article and in its online supporting data.

## Supporting Information Available

Characteristics of the *Shigella* and STEC AB_5_ Shiga toxins (Table S1); structure of the STEC AB5 toxin Shiga toxin and characteristics of Stx1 and Stx2 A and B subunits (Figure S1); statistics of the Stx enzyme activity assay for STEC and controls (Figure S2); optimal assay conditions for Stx detection in culture supernatants (Figure S3); statistics of Vero cell cytotoxicity assay and Stx activity assay for Stx1 and Stx2 subtypes (see Figure 4A) (Figure S4); statistics of Vero cell cytotoxicity assay and Stx activity assay for different *Shigella* strains (Figure S5); quality control analysis report of StxSense 1 (biomers.net) (Figure S6); quality control analysis report of StxSense 2 (integrated DNA Technologies, idt) (Figure S7); quality control analysis report of StxSense 3 (integrated DNA Technologies, idt) (Figure S8); and quality control analysis report of StxSense 4 (integrated DNA Technologies, idt) (Figure S9) (PDF)
STEC strains used in the study (Table S2); other intestinal *E. coli* pathovar strains without Stx used in the study (Table S3); *Yersinia* and *Salmonella* strains used in the study (Table S4); and *Shigella* strains used in the study (Table S5) (XLSX)
