# Supplementary material for: Rapid Enzymatic Detection of Shiga-Toxin-Producing E. coli Using Fluorescence-Labeled Oligonucleotide Substrates
Source: ACS Infect Dis. 2024 Nov 22;10(12):4103–14. doi: 10.1021/acsinfecdis.4c00221 (PMC11650650; doi:10.1021/acsinfecdis.4c00221)
Supplement: Supplementary file 1 — id4c00221_si_001.pdf [file id4c00221_si_001.pdf]

Supporting Information

Rapid enzymatic detection of Shigatoxin-producing *E. coli* using fluorescence-labeled oligonucleotide substrates

Isabell Ramming1, Christina Lang1, Samuel Hauf1, Maren Krüger3, Sylvia Worbs3, Carsten Peukert2, Angelika Fruth1, Brigitte G. Dorner3, Mark Brönstrup2,4, Antje Flieger1*

1 Robert Koch Institute, Department for Infectious Diseases, Division of Enteropathogenic Bacteria and Legionella (FG11), National Reference Centre for Salmonella and other Enteric Bacterial Pathogens, 38855 Wernigerode, Germany

2 Helmholtz Centre for Infection Research, Department of Chemical Biology (CBIO), 38124 Braunschweig, Germany

3 Robert Koch Institute, Centre for Biological Threats and Special Pathogens, Biological Toxins (ZBS3), 13353 Berlin, Germany

4 German Center for Infection Research (DZIF), Site Hannover-Braunschweig, 38124 Braunschweig, Germany

* Corresponding author: Prof. Dr. Antje Flieger fliegera@rki.de (AF)

Co-authors:
Dr. Christina Lang, langc@rki.de; Dr. Samuel Hauf, samuel.hauf2@oist.jp ; Dr. Maren Krüger, KruegerM@rki.de; Dr. Sylvia Worbs, worbss@rki.de; Dr. Carsten Peukert, c.peukert@hotmail.de; Dr. Angelika Fruth, frutha@rki.de; Dr. Brigitte G. Dorner, dornerb@rki.de; Prof. Dr. Mark Brönstrup, mark.broenstrup@helmholtz-hzi.de; Prof. Dr. Antje Flieger, fliegera@rki.de

Ramming et al., Table S1

Tab. S1: Characteristics of the *Shigella* and STEC AB_5 Shiga toxins

	Stx	Stx1	Stx2	Reference
**Organism**	*Shigella dysenteriae*	STEC	STEC	
**Subtypes**	1a	1a, 1c, 1d	2a-o	[1] [2]
**Protein sequence identity to Stx of *S. dysenteriae***	100 %	91 – 99 %*	~ 55 %*	[3]
**No. of amino acids**		A1: 251<br>A2: 42	A1: 250<br>A2: 47	[4]
**Active site in StxA1**	Glu 167	Glu 167 covered by the A2 chain in the holotoxin (A1-A2)	Glu 166 open conformation in the holotoxin (A1-A2)	[5] [6] [7]
**Bacterial growth media and Stx production**	n.k.	Culture media affect bacterial growth and concentration of Stx in supernatants		[8]
**Activity of StxA1 on SRL RNA**	n.k.	k_{cat} = 21.5 min^{-1}; lower compared to Stx2A1	k_{cat} = 62.6 min^{-1}; higher compared to Stx1A1	[9]
**Optimal pH for activity**	n.k.	*in vitro*: acidic pH (pH 4.5)		[9]
**Activity and temperature**	Activity loss ≥ 65°C	Activity loss ≥ 65°C	Activity loss ≥ 85°C	[10]

* Sequence identities of all Stx subtypes are summarized in Bergan *et al.,* 2012; n.k., not known

Ramming et al., Figure S1

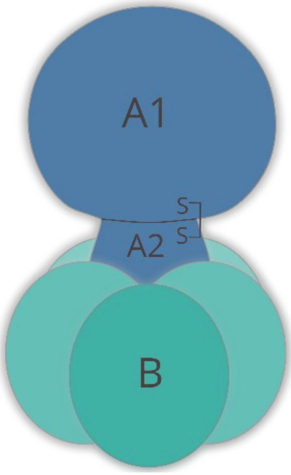

Sub-unit	MW [kDa]	No. of amino acids (without signal peptide)		Function	Reference
		Stx1	Stx2		
**A**	32	293	297	A1 and A2 connected by disulfide bridge after cleavage by furin	1, 11
**A1**	27.5	251	250	Enzymatic activity, 28S rRNA *N*-glycosidase activity	1
**A2**	4.5	42	47	Indispensable for holotoxin structure	12
**B**	7.7	69	68–70	Binding to Gb3/CD77 receptor	13

Fig. S1: Structure of the STEC AB5 toxin Shiga toxin (Bergan et al., 2012) and characteristics of Stx1 and Stx2 A and B subunits.

Ramming et al., Figure S2: SRL substrates (belongs to Fig. 2)

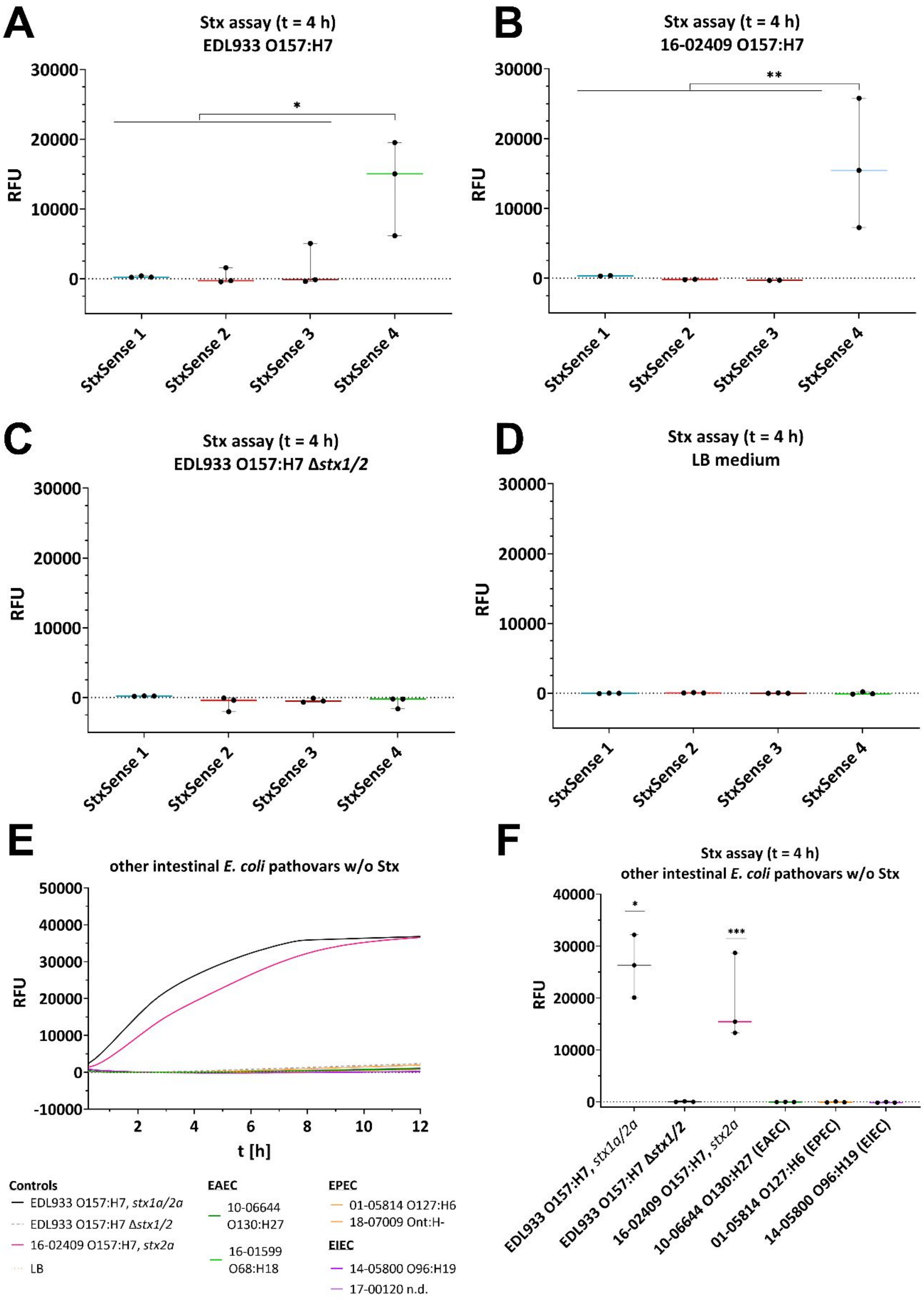

Ramming et al., Figure S2 (continued)

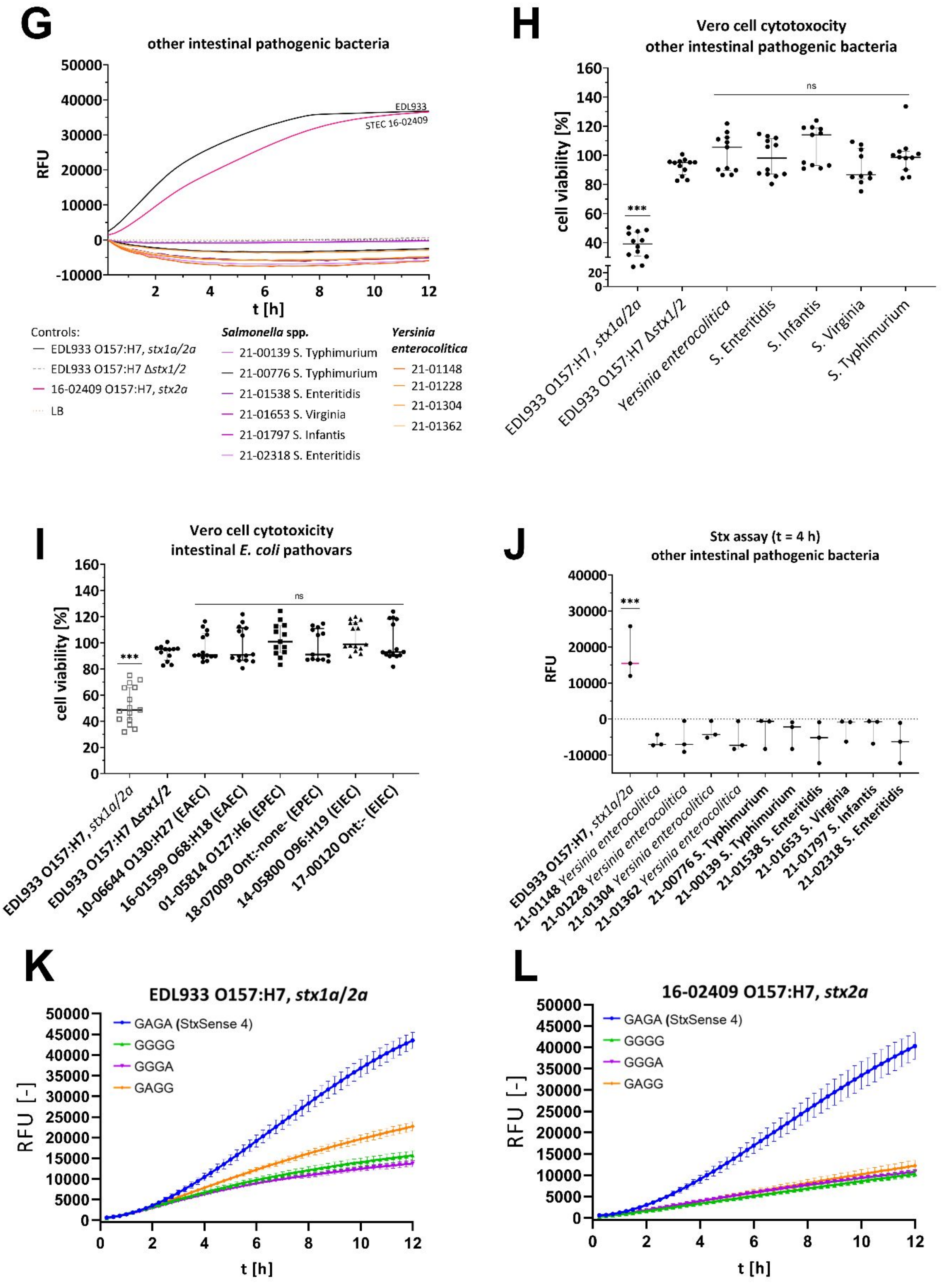

Ramming et al., Figure S2 (continued)

M

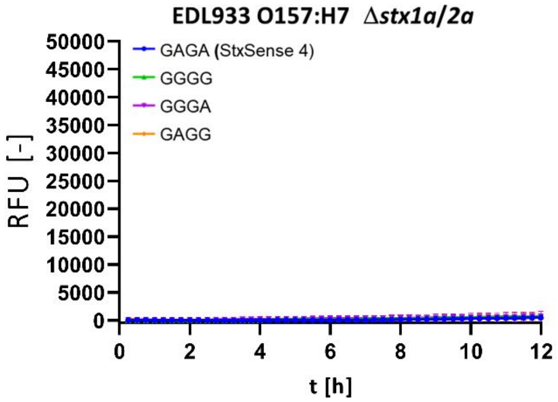

N

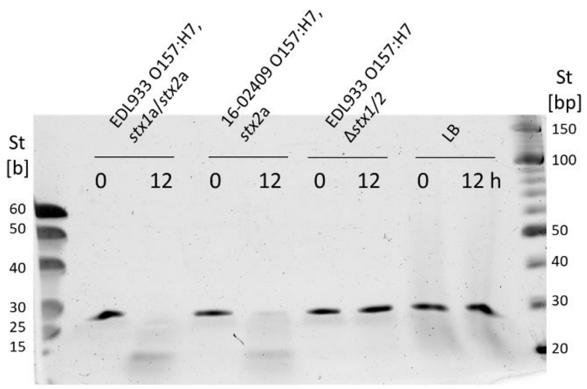

Fig. S2: Statistics of the Stx enzyme activity assay for STEC and controls. Statistics of the Vero cell cytotoxicity and Stx enzyme activity assay for other intestinal pathogenic bacteria producing no Stx. Analysis of StxSense 4-derived substrates with modified GAGA recognition sequence and verification of StxSense 4 substrate cleavage. Detected fluorescence as a marker of substrate hydrolysis by Stx from different culture supernatants after 4 h incubation of **(A)** EDL933 O157:H7, *stx1a/2a*; **(B)** 16-02409 O157:H7, *stx2a*, **(C)** EDL933 O157:H7 Δ*stx1/2*, and **(D)** LB using the four SRL substrates. Data refer to the data shown in Fig. 2. Other intestinal *E. coli* pathovars: **(E and F)** Stx enzymatic activity assay for culture supernatants for up to 12 h (E) or for 4 h (F) and **(I)** Vero cell cytotoxicity assay. Other intestinal pathogenic bacteria: **(G and H)** Stx enzymatic activity assay for culture supernatants for up to 12 h (H) or for 4 h (I) and (J) Vero cell cytotoxicity assay. **(K, L, M)** Analysis of Stx activity from culture supernatants of STEC strains EDL933 **(K)** and 16-02409 **(L)** and negative control EDL933 Δ*stx1/2* **(M)** using SRL substrate StxSense 4 containing the GAGA motif and further substrates with modified recognition site, such as GGGG, GGGA and GAGG. (N) Verification of StxSense 4 cleavage after incubation with culture supernatants of STEC strains EDL933 and 16-02409 and negative controls EDL933 Δ*stx1/2* or LB broth. The experiment was carried out comparable to the enzyme activity assay. At different time points (0 h, 12 h reaction time), 15 µL of the sample was taken and separated on a 15 % UREA PAGE gel and stained with GelRed. The IDT-Oligo Length Standard 10/60 ladder (left side) and the O'RangeRuler 10bp DNA ladder (right side) were used as standard. For the Vero cell cytotoxicity assay (three experiments each n=3), cell viability of Vero cells was analyzed using MTT assay after inoculation with diluted bacterial culture supernatants (1:400) for 48 h**.** Symbols represent the different strains and data points. For statistics of Stx enzymatic activity assay, RFU at 4 h reaction time and 44 °C are shown. The results of the Stx enzyme activity assay represent the medians of triplicate samples (n=3) and are representative of three independent experiments. Error bars represent standard deviation. Statistical analysis was performed by unpaired, double-sided t test (A-D, F, H), Mann-Whitney test (I and J) (*, $p < 0.05$; **, $p < 0.01$; ***, $p < 0.001$), with results compared to those of EDL933 O157:H7 Δ*stx1/2*. RFU, Relative Fluorescent Unit; ns, not significant.

Ramming et al., Figure S3: assay conditions

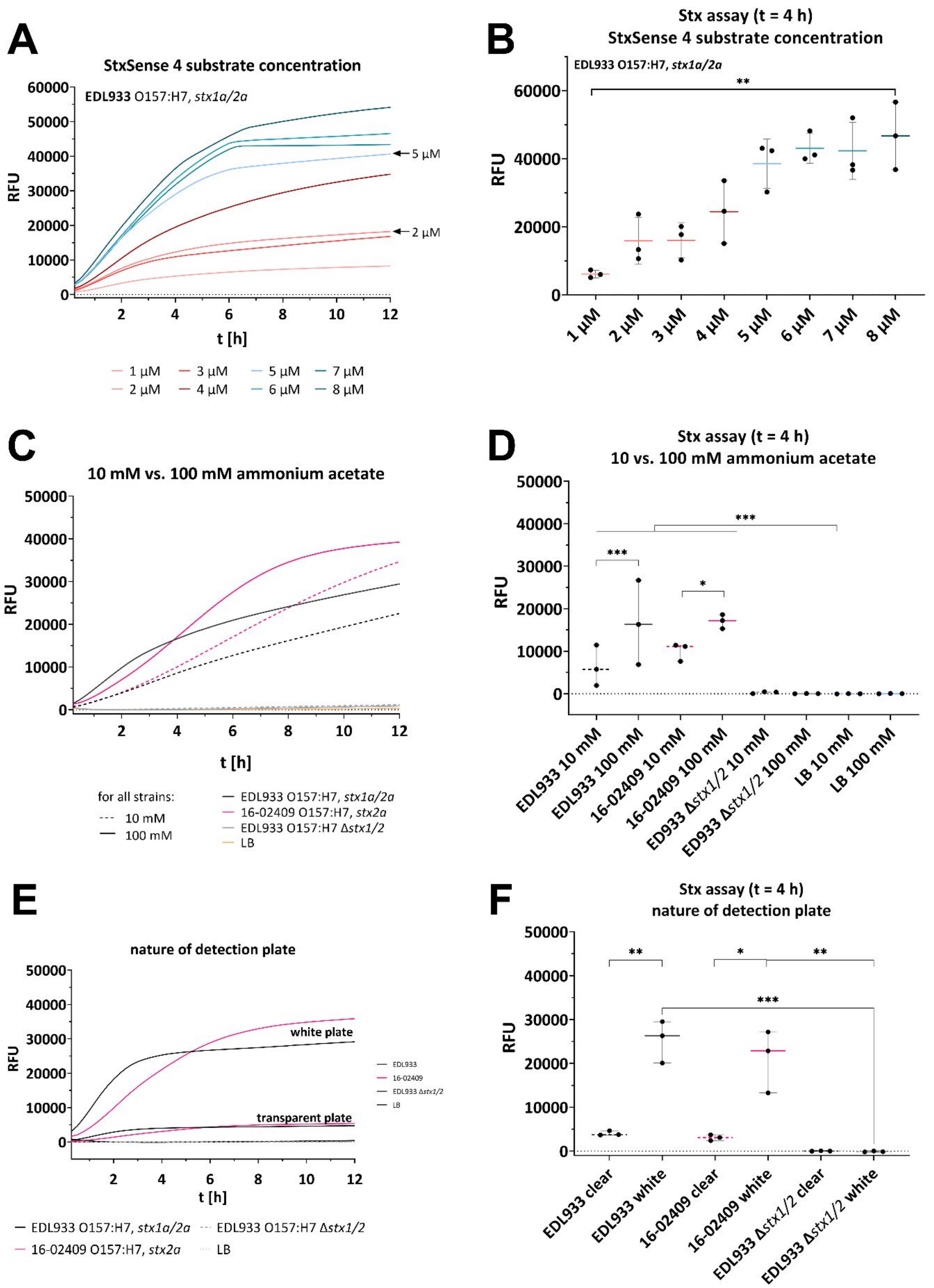

Ramming et al., Figure S3 (continued)

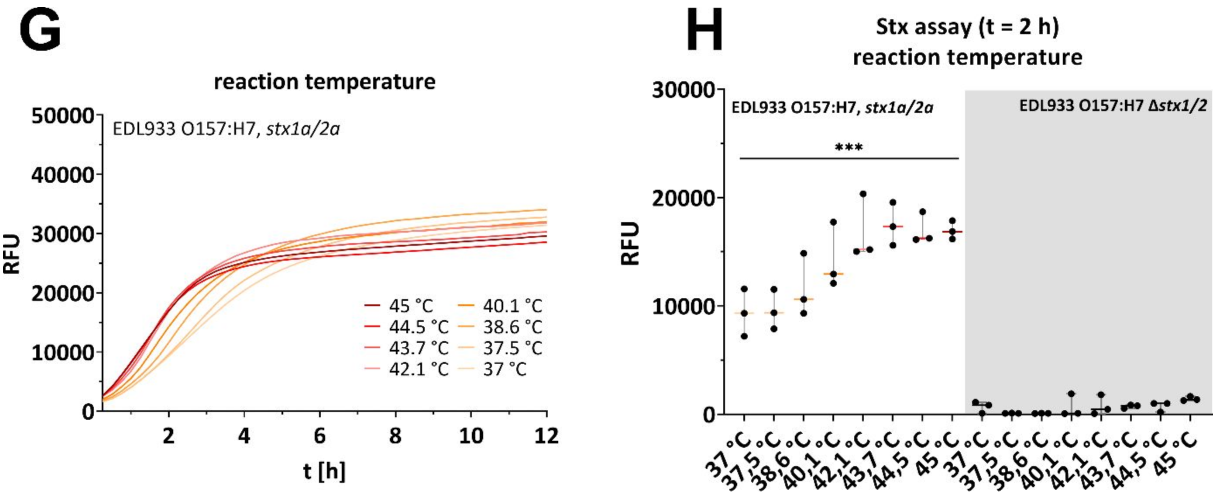

Fig. S3: Optimal assay conditions for Stx detection in culture supernatants. Detected fluorescence as a marker of substrate hydrolysis by Stx from positive control EDL933 O157:H7, *stx1a/2a* and/or test strain STEC 16-02409 O157:H7, *stx2a*, and negative control EDL933 O157:H7 Δ*stx1/2*. **(A and B)** Optimal fluorescence readout for Stx was achieved using SRL substrate StxSense 4 concentrations from 2 µM in 100 mM ammonium acetate. **(C and D)** 100 mM ammonium acetate yielded in higher fluorescence signals *stx2a*-producing strain compared to 10 mM ammonium acetate. **(E and F)** Using a white 96 well plate instead of a clear plate was essential for fluorescence detection of Stx-positive samples. **(G and H)** Reaction temperatures above 43.7 °C were optimal Stx detection. Reaction conditions for (A, B, E, F) were 44 °C, 100 mM ammonium acetate, 2 µM StxSense 4. The results represent the medians of triplicate samples (n = 3) and are representative of three independent experiments. Error bars represent standard deviation. Statistical analysis was performed by Mann-Whitney test for non-normally distributed samples (B, H) and unpaired, double-sided t test (D, F) (*, $p < 0.05$; **, $p < 0.01$; ***, $p < 0.001$), with results compared to those of EDL933 O157:H7 Δ*stx1/2* (negative control; -- [grey dashed lines]). RFU, relative fluorescence units; t [h], time [hours].

Ramming et al., Figure S4: Stx-producing strains (belongs to Fig. 4)

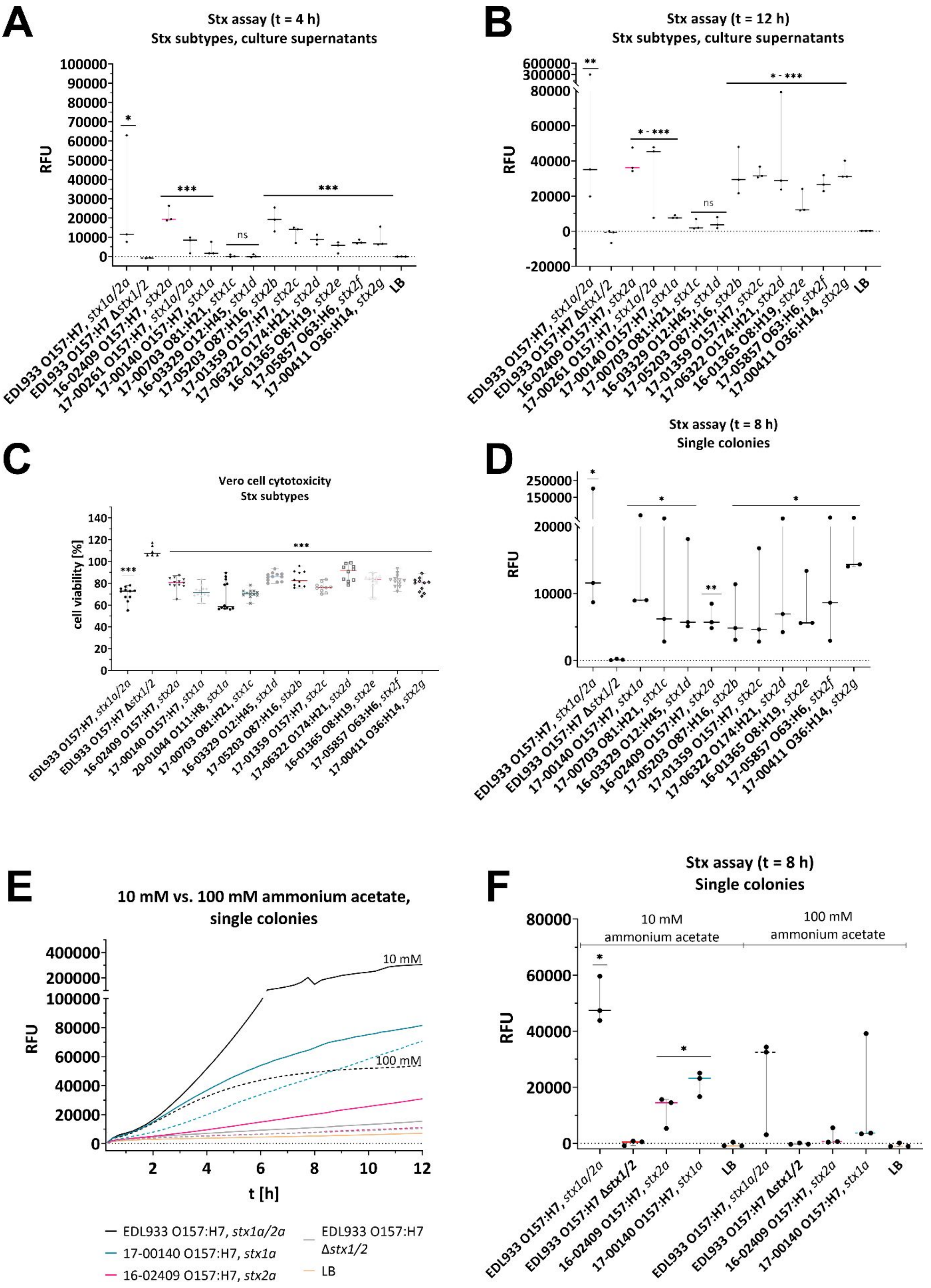

Ramming et al., Figure S4 (continued)

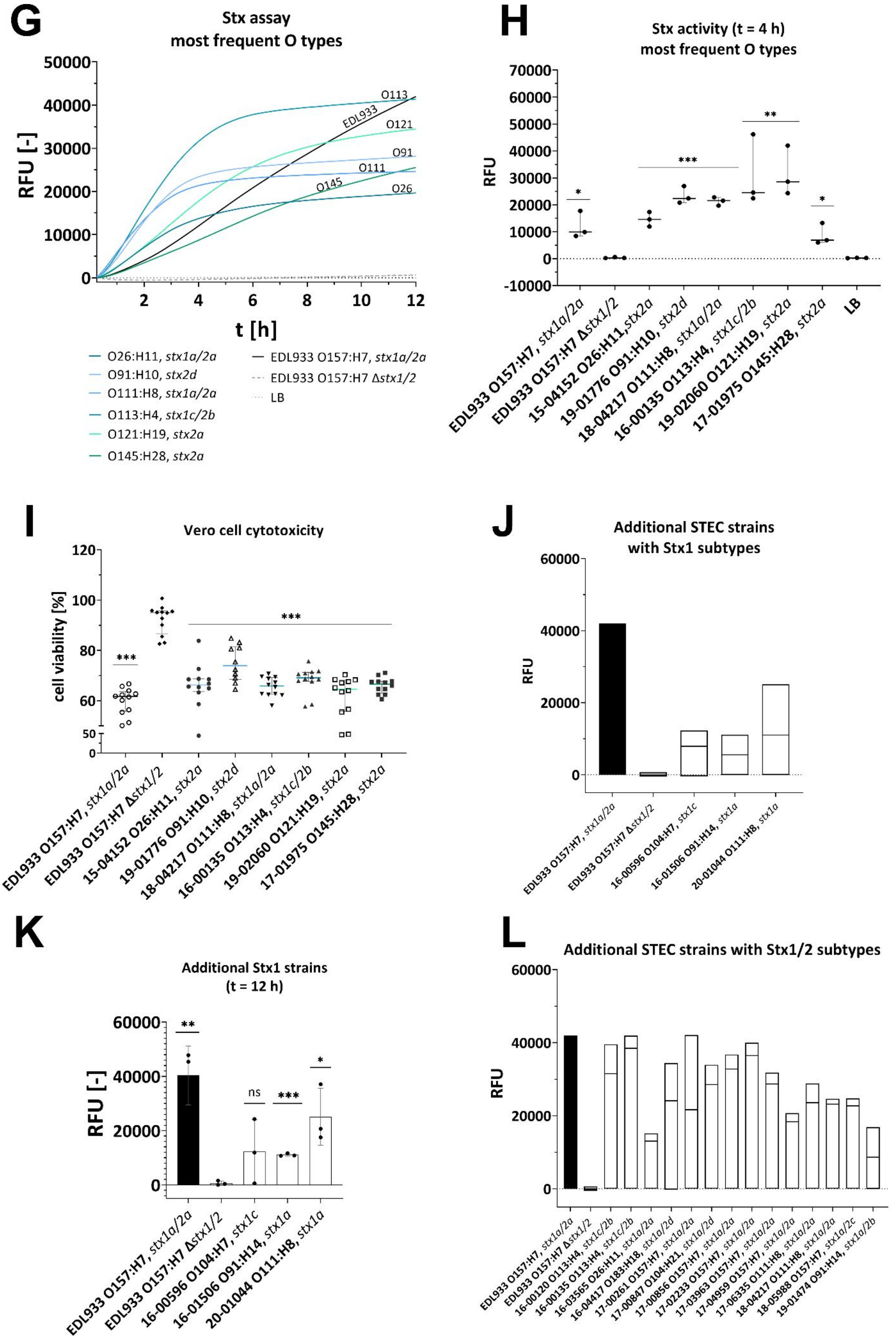

Ramming et al., Figure S4 (continued)

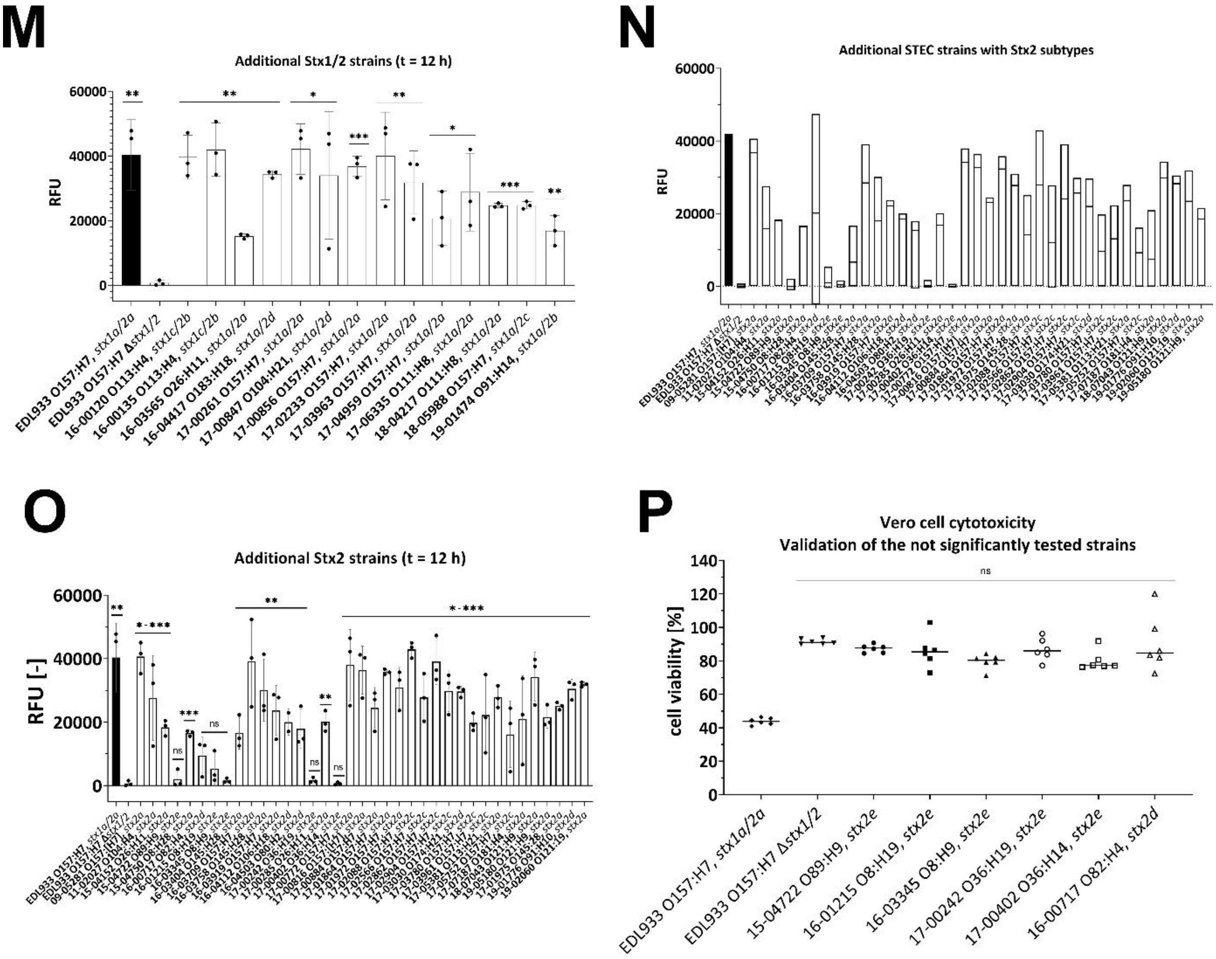

Fig. S4: Statistics of Vero cell cytotoxicity assay and Stx activity assay for Stx1 and Stx2 subtypes (see Fig. 4A). (A, B, D-H, J-O) Detected fluorescence of Stx activity from **(A and B)** culture supernatants of STEC strains covering Stx1a-Stx1d and Stx2a-2g at (A) 4 h and (B) 12 h; **(D)** single colonies of STEC strains covering Stx1a-Stx1d and Stx2a-2g, **(E and F)** single colonies of STEC strains and influence of 10 mM vs. 100 mM ammonium acetate, **(G and H)** most frequent serotypes; **(J to O)** Detected fluorescence of culture supernatants of different STEC strains comprising **(J and K)** Stx1, **(L and M)** Stx1/2, **(N and O)** Stx2, with positive control EDL933 O157:H7, *stx1a/2a* (black filled bar), and negative control EDL933 O157:H7 Δ*stx1/2* (grey filled bar) as floating bars (min to max RFU) with line at the median RFU over 12 h and statistics. (J to O) were performed within the same analysis. Shown are the RFU of culture supernatants at 4 h reaction time in 100 mM ammonium acetate, if not stated otherwise, 2 µM StxSense 4, 44 °C, white reaction plate. **(C, I and P)** Cell viability of Vero cells was analyzed using MTT assay after inoculation with diluted bacterial culture supernatants (1:400) for 48 h: **(C)** STEC strains comprising different Stx subtypes, **(I)** most frequent serotypes. Data refer to the data shown in Fig. 4, **(P)** Verification of STEC strains not significantly tested within the STEC detection assay. The results are medians of triplicate samples (n = 3) of two to three independent experiments. Error bars represent standard deviation. Statistical analysis was performed by Mann-Whitney test (A, B, D-F, H, K, M, O) and unpaired, double-sided t test (C, I) (*, $p < 0.05$; **, $p < 0.01$; ***, $p < 0.001$), with results compared to those of EDL933 O157:H7 Δ*stx1/2*. RFU, Relative Fluorescent Unit; ns, not significant.

Ramming et al., Figure S5: *Shigella*

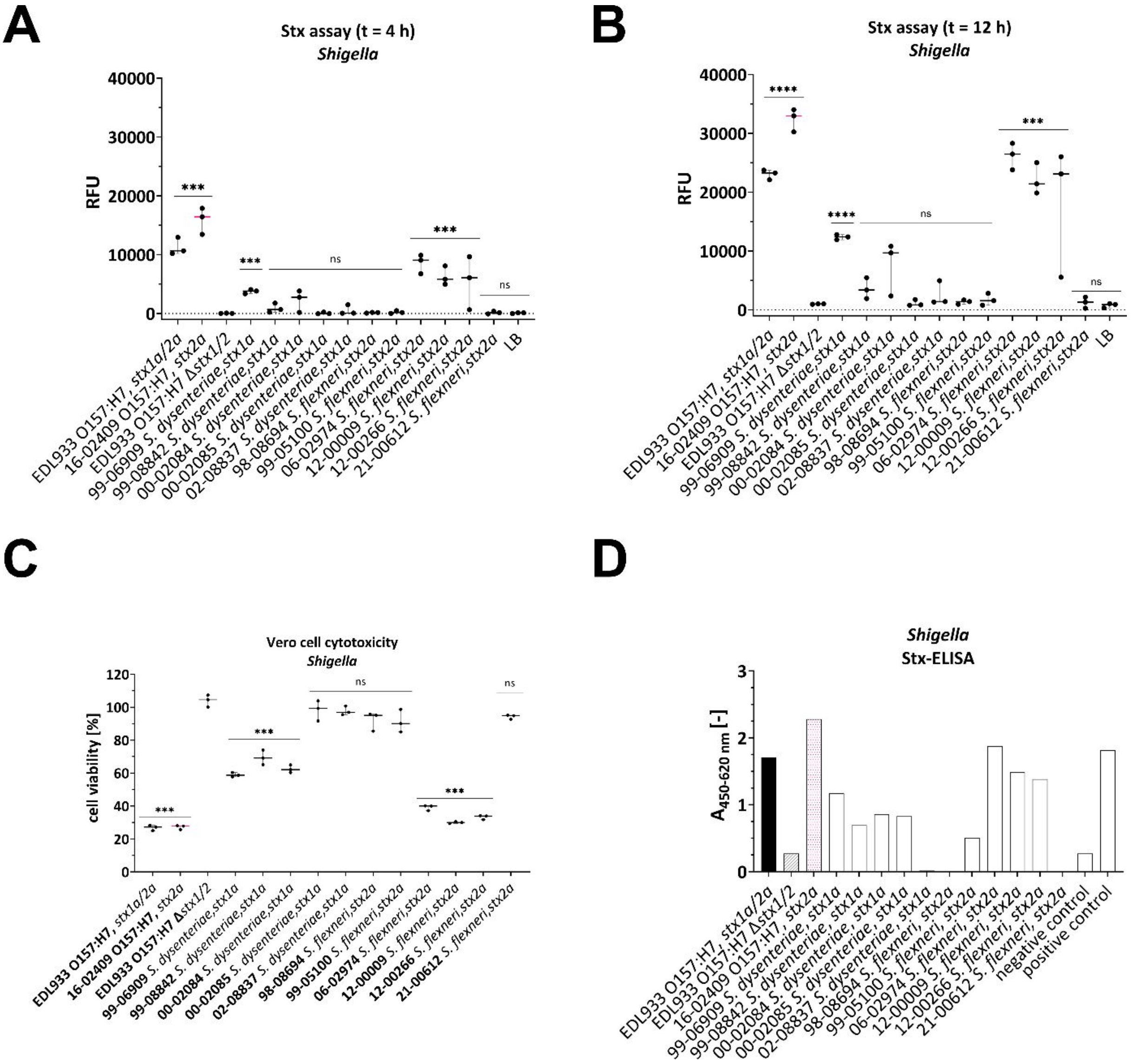

Fig. S5: Statistics of Vero cell cytotoxicity assay and Stx activity assay for different *Shigella* strains.
(A and B) Detected fluorescence of culture supernatants of different *Shigella* spp. strains and positive control EDL933 O157:H7, *stx1a/2a*, STEC strain 16-02409 O157:H7, *stx2a* and negative control EDL933 O157:H7 Δ*stx1/2* at (A) 4h and (B) 12 h. The assay was performed using 100 mM ammonium acetate, 2 µM StxSense 4, 44 °C. **(C)** Cell viability of Vero cells was analyzed using MTT assay after inoculation with diluted bacterial culture supernatants (1:400) for 48 h. **(D)** Commercial Stx ELISA (R-Biopharm AG, Darmstadt, Germany) of culture supernatant (OD_{600} of 3.0) from different *Shigella* strains detecting Stx1 and Stx2 (n = 1). All other results are medians of triplicate samples (n = 3) of two to three independent experiments. Error bars represent standard deviation. Statistical analysis was performed by Mann-Whitney test (A) and unpaired, double-sided t test (B) (*, $p < 0.05$; **, $p < 0.01$; ***, $p < 0.001$) with results compared to those of EDL933 O157:H7 Δ*stx1/2*. RFU, Relative Fluorescent Unit; ns, not significant.

Ramming et al., Figure S6

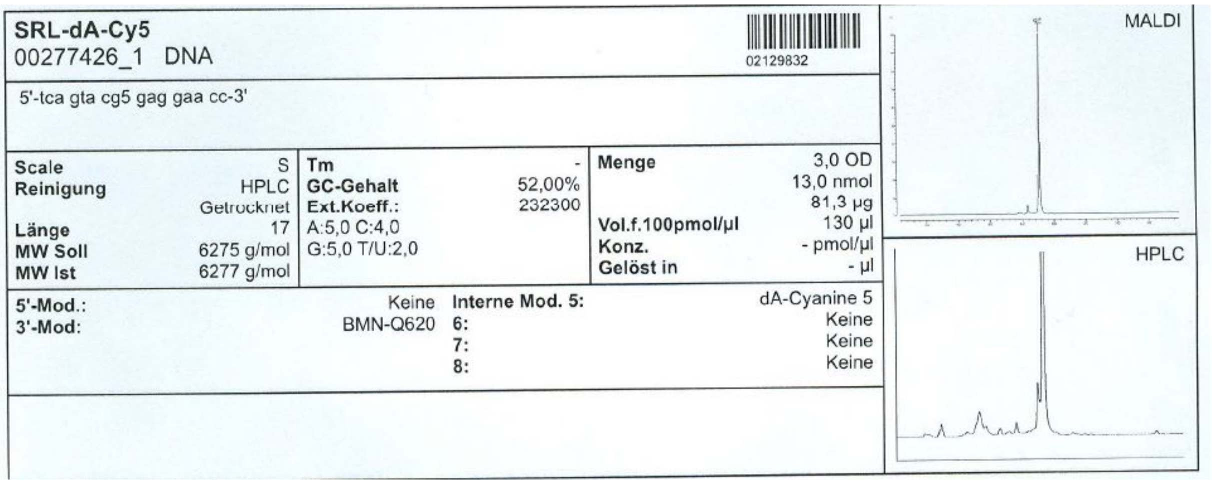

SRL-dA-Cy5
00277426_1 DNA

02129832

5'-tca gta cg5 gag gaa cc-3'

**Scale**	S	**Tm**	-	**Menge**	3,0 OD
**Reinigung**	HPLC	**GC-Gehalt**	52,00%		13,0 nmol
	Getrocknet	**Ext.Koeff.:**	232300		81,3 µg
**Länge**	17	A:5,0 C:4,0		**Vol.f.100pmol/µl**	130 µl
**MW Soll**	6275 g/mol	G:5,0 T/U:2,0		**Konz.**	- pmol/µl
**MW Ist**	6277 g/mol			**Gelöst in**	- µl

**5'-Mod.:**	Keine	**Interne Mod. 5:**	dA-Cyanine 5
**3'-Mod:**	BMN-Q620	**6:**	Keine
		**7:**	Keine
		**8:**	Keine

Fig. S6: Quality control analysis report of StxSense 1 (biomers.net).

Ramming et al., Figure S7

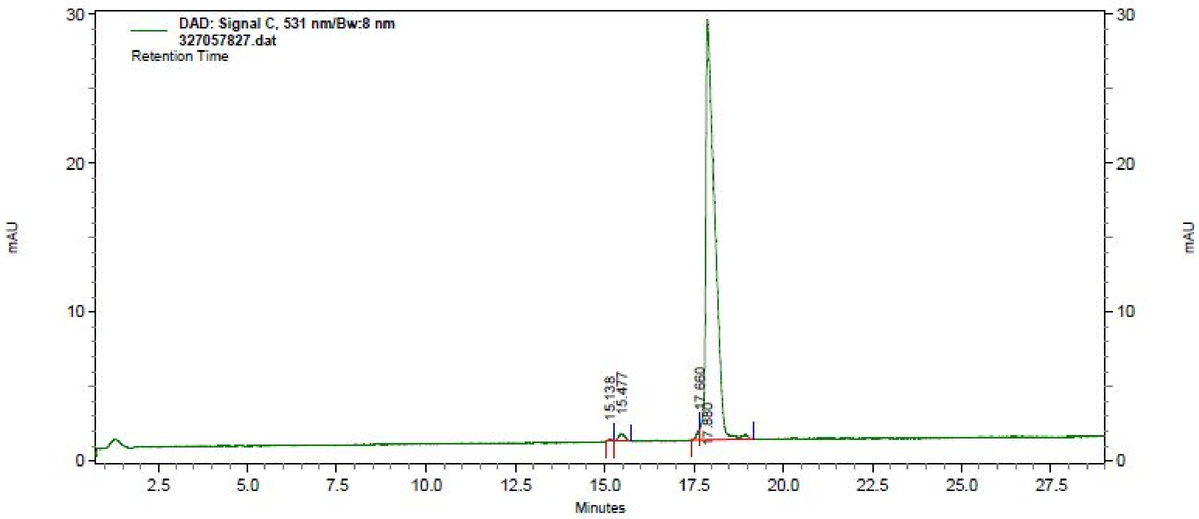

DAD: Signal C, 531 nm/Bw:8 nm Results

Peak #	Time	Height	Area	Area Percent
1	15.14	293	2387	0.2
2	15.48	1108	14277	1.3
3	17.66	1460	7702	0.7
4	17.88	59110	1033939	97.7
Totals		61971	1058305	100.0

Fig. S7: Quality control analysis report of StxSense 2 (integrated DNA Technologies, idt).

Ramming et al., S8 Figure

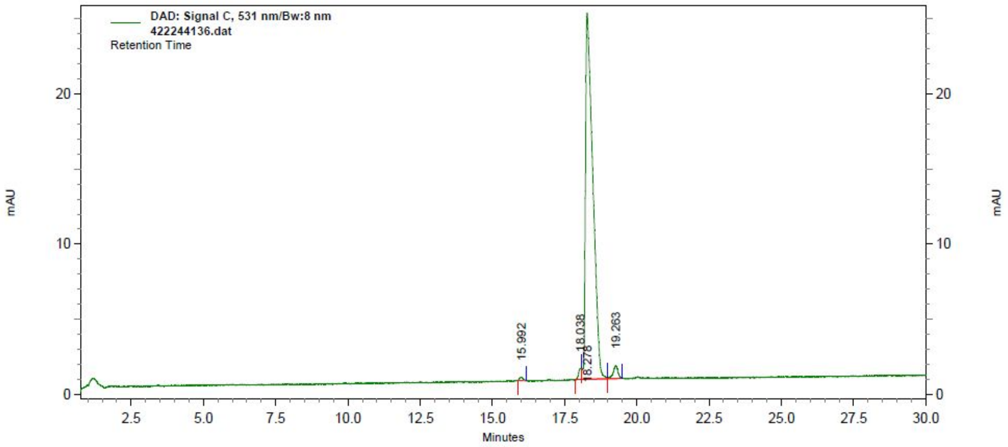

DAD: Signal C, 531 nm/Bw:8 nm Results

Peak #	Time	Height	Area	Area Percent
1	15.99	483	4016	0.4
2	18.04	1597	10996	1.2
3	18.28	51150	900948	96.2
4	19.26	1827	20544	2.2
Totals		55057	936504	100.0

S8 Fig. Quality control analysis report of StxSense 3 (integrated DNA Technologies, idt).

Ramming et al., S9 Figure

Integrated DNA Technologies Page 1 of 1

Analytical OligoPro CE Report

Sample ID: 233071385-21 SS HPLC A2 55887
Instrument: Oligo Pro (Offline) Operator: HTA
Acquired: 8/22/2022 5:55:00 AM Reviewed: 8/22/2022 7:48:16 AM

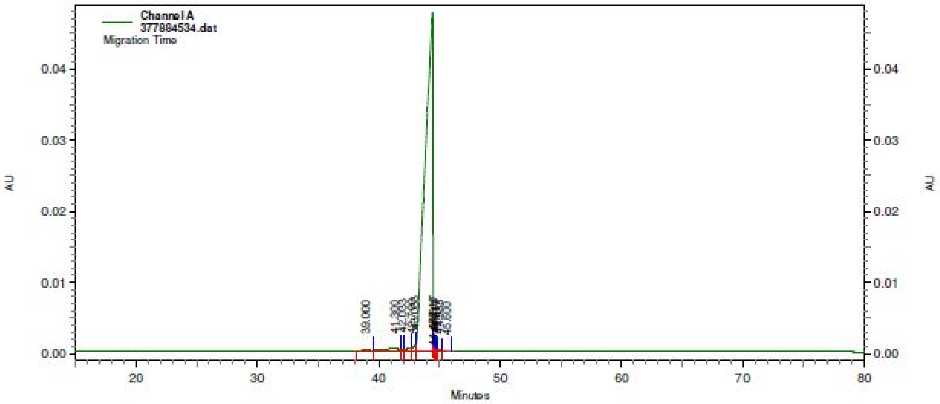

Channel A Results

Peak #	Time	Height	Corrected Area	Corrected Area Percent
1	39.00	145	160	0.4
2	41.30	372	757	1.7
3	42.03	284	92	0.2
4	42.70	456	294	0.7
5	43.03	821	258	0.6
6	44.43	47506	42884	95.9
7	44.52	879	50	0.1
8	44.62	433	45	0.1
9	44.72	292	43	0.1
10	44.98	269	86	0.2
11	45.60	70	31	0.1
Totals		51527	44700	100.0

S9 Fig. Quality control analysis report of StxSense 4 (integrated DNA Technologies, idt).

Supplemental Methods

Vero cell cytotoxicity assay. Toxicity of Stx towards Vero cells was determined as described previously [14] with modifications. Prior to addition of culture supernatants, 1x10^5 Vero cells/mL were seeded into a 96 well-plate (Greiner Bio-One; Kremsmünster, Austria) and were incubated for 24h at 37 °C and 5 % CO_2. Culture supernatants were diluted 1:400 in DMEM with 10 % FBS and 200 µL were added to each well and incubated for 48 h at 37 °C and under 5 % CO_2. After incubation, the cells were washed with 1× PBS and 100 µL 1-(4,5-Dimethylthiazol-2-yl)-3,5-diphenylformazan (MTT; 0.5 mg/mL in PBS; Sigma) were added for 1 h. After removal, 100 µL acidified isopropanol (isopropanol with 4 % v/v HCl, 32 %) were added. The absorption at 570 nm was photometrically measured at 570 nm (Tecan Infinite M-1000 Pro; Tecan, Suisse) [15]. The proportion of damaged to viable cells (viability, in %) was calculated using mean absorbance of sample/mean absorbance of Vero cell control × 100.

SRL substrate cleavage analysis by means of UREA-PAGE. 14.6 µl 100 mM depurination buffer, 5 µl culture supernatant and 0,4 µl StxSense 4 were mixed and incubated in a thermo mixer at 44 °C. After 0 h and 12 h 5 µl of loading dye were added. The entire reaction mix was then transferred to a 15 % UREA-polyacrylamide gel and separated at 150 V for 2 h [16]. The gel was subsequently stained with GelRed solution for 5 min (Merck Millipore; Darmstadt, Germany) and the cleavage products were detected in ChemiDoc GelRed filter. The IDT-Oligo Length Standard 10/60 Ladder and the O'RangeRuler 10bp DNA Ladder (Thermo Scientific) were used as standards.

References of supplemental data

1. Scheutz, F.; Teel, L. D.; Beutin, L.; Piérard, D.; Buvens, G.; Karch, H.; Mellmann, A.; Caprioli, A.; Tozzoli, R.; Morabito, S.; Strockbine, N. A.; Melton-Celsa, A. R.; Sanchez, M.; Persson, S.; O'Brien, A. D., Multicenter evaluation of a sequence-based protocol for subtyping Shiga toxins and standardizing Stx nomenclature. *J. Clin. Microbiol.* **2012,** *50*, 2951-2963.
2. Yang, X.; Liu, Q.; Sun, H.; Xiong, Y.; Matussek, A.; Bai, X., Genomic Characterization of Escherichia coli O8 Strains Producing Shiga Toxin 2l Subtype. *Microorganisms* **2022,** *10*, 1245.
3. Bergan, J.; Dyve Lingelem, A. B.; Simm, R.; Skotland, T.; Sandvig, K., Shiga toxins. *Toxicon* **2012,** *60* (6), 1085-107.
4. Li, X. P.; Tumer, N. E., Differences in Ribosome Binding and Sarcin/Ricin Loop Depurination by Shiga and Ricin Holotoxins. *Toxins* **2017,** *9* (4), 133.
5. Yamasaki, S.; Furutani, M.; Ito, K.; Igarashi, K.; Nishibuchi, M.; Takeda, Y., Importance of arginine at position 170 of the A subunit of Vero toxin 1 produced by enterohemorrhagic Escherichia coli for toxin activity. *Microb Pathog* **1991,** *11* (1), 1-9.
6. Steyert, S. R.; Sahl, J. W.; Fraser, C. M.; Teel, L. D.; Scheutz, F.; Rasko, D. A., Comparative genomics and stx phage characterization of LEE-negative Shiga toxin-producing Escherichia coli. *Front Cell Infect Microbiol* **2012,** *2*, 133.
7. Jackson, M. P., Structure-function analyses of Shiga toxin and the Shiga-like toxins. *Microb Pathog* **1990,** *8* (4), 235-42.
8. Rocha, L. B.; Piazza, R. M., Production of Shiga toxin by Shiga toxin-expressing Escherichia coli (STEC) in broth media: from divergence to definition. *Lett Appl Microbiol* **2007,** *45* (4), 411-7.
9. Basu, D.; Li, X. P.; Kahn, J. N.; May, K. L.; Kahn, P. C.; Tumer, N. E., The A1 subunit of Shiga toxin 2 has higher affinity for ribosomes and higher catalytic activity than the A1 subunit of Shiga toxin 1. *Infec. Imm.* **2015,** *84*, 149-161.
10. Chan, Y. S.; Ng, T. B., Shiga toxins: from structure and mechanism to applications. *Appl Microbiol Biotechnol* **2016,** *100* (4), 1597-1610.
11. Gyles, C. L.; De Grandis, S. A.; MacKenzie, C.; Brunton, J. L., Cloning and nucleotide sequence analysis of the genes determining verocytotoxin production in a porcine edema disease isolate of Escherichia coli. *Microb Pathog* **1988,** *5* (6), 419-26.
12. Menge, C., Molecular biology of escherichia coli shiga toxins' effects on mammalian cells. *Toxins* **2020,** *12*.
13. Takeda, Y.; Kurazono, H.; Yamasaki, S., Vero toxins (Shiga-like toxins) produced by enterohemorrhagic Escherichia coli (verocytotoxin-producing E. coli). *Microbiol Immunol* **1993,** *37* (8), 591-9.
14. Roberts, P. H.; Davis, K. C.; Garstka, W. R.; Bhunia, A. K., Lactate dehydrogenase release assay from Vero cells to distinguish verotoxin producing Escherichia coli from non-verotoxin producing strains. *J Microbiol Methods* **2001,** *43* (3), 171-81.
15. Mosmann, T., Rapid colorimetric assay for cellular growth and survival: application to proliferation and cytotoxicity assays. *J Immunol Methods* **1983,** *65* (1-2), 55-63.
16. Summer, H.; Gramer, R.; Droge, P., Denaturing urea polyacrylamide gel electrophoresis (Urea PAGE). *J Vis Exp* **2009,** (32).

Tab. S2-S5 (Excel file with sub tables)

Frank, C., Werber, D., Cramer, J. P., Askar, M., Faber, M., an der Heiden, M., Bernard, H., Fruth, A., Prager, R., Spode, A., Wadl, M., Zoufaly, A., Jordan, S., Kemper, M. J., Follin, P., Müller, L., King, L. A., Rosner, B., Buchholz, U., … Krause, G. (2011). Epidemic Profile of Shiga-Toxin–Producing Escherichia coli O104:H4 Outbreak in Germany. *New England Journal of Medicine*, *365*(19), 1771–1780.

Gobert, A. P., Vareille, M., Glasser, A.-L., Hindré, T., De Sablet, T., & Martin, C. (2007). Shiga Toxin Produced by Enterohemorrhagic Escherichia coli Inhibits PI3K/NF-B Signaling Pathway in Globotriaosylceramide-3-Negative Human Intestinal Epithelial Cells 1. *The Journal of Immunology*, *178*, 8168–8174.

Lang, C., Hiller, M., Konrad, R., Fruth, A., & Flieger, A. (2019). Whole-Genome-Based Public Health Surveillance of Less Common Shiga Toxin-Producing Escherichia coli Serovars and Untypeable Strains Identifies Four Novel O Genotypes. *Journal of Clinical Microbiology*, *57*(10).

Lang, C., Fruth, A., Campbell, I. W., Jenkins, C., Smith, P., Weill, F.-X., Nübel, U., Grad, Y. H., & Flieger, A. (2023). O-antigen diversification masks identification of highly pathogenic STEC O104:H4-like 1 strains 2 3. *Microbiology Spectrum*

Perna, N. T., Plunkett, G., Burland, V., Mau, B., Glasner, J. D., Rose, D. J., Mayhew, G. F., Evans, P. S., Gregor, J., Kirkpatrick, H. A., Pósfai, G., Hackett, J., Klink, S., Boutin, A., Shao, Y., Miller, L., Grotbeck, E. J., Davis, N. W., Lim, A., Blattner, F. R. (2001). Genome sequence of enterohaemorrhagic Escherichia coli O157:H7. *Nature*, *409*(6819), 529–533.

Scheutz, F., L. D. Teel, L. Beutin, D. Piérard, G. Buvens, H. Karch, A. Mellmann, A. Caprioli, R. Tozzoli, S. Morabito, N. A. Strockbine, A. R. Melton-Celsa, M. Sanchez, S. Persson and A. D. O'Brien (2012). Multicenter evaluation of a sequence-based protocol for subtyping Shiga toxins and standardizing Stx nomenclature. Journal of Clinical Microbiology. 50: 2951-2963.

Schmidt, H., Russmann, H., & Karch, H. (1993). Virulence determinants in nontoxinogenic Escherichia coli O157 strains that cause infantile diarrhea. *Infection and Immunity*, *61*(11), 4894–4898.
